# Development and validation of a deep learning-based pathomics signature for prognosis and chemotherapy benefits in colorectal cancer: a retrospective multicenter cohort study

**DOI:** 10.3389/fimmu.2025.1602909

**Published:** 2025-07-08

**Authors:** Shenghan Lou, Yanming Huang, Fenqi Du, Jingmin Xue, Genshen Mo, Hao Li, Zhanjiang Yu, Yuanchun Li, Hang Wang, Yuze Huang, Haonan Xie, Wenjie Song, Xinyue Zhang, Huiying Li, Chun Lou, Peng Han

**Affiliations:** ^1^ Department of Oncology Surgery, Harbin Medical University Cancer Hospital, Harbin, Heilongjiang, China; ^2^ Department of General Surgery, The Third Affiliated Hospital of Qiqihar Medical University, Qiqihar, Heilongjiang, China; ^3^ Department of General Surgery, The Second Affiliated Hospital of Qiqihar Medical University, Qiqihar, Heilongjiang, China; ^4^ Department of Breast Surgery, Harbin Medical University Cancer Hospital, Harbin, Heilongjiang, China; ^5^ Department of Pathology, Harbin Medical University Cancer Hospital, Harbin, Heilongjiang, China; ^6^ Heilongjiang Province Key Laboratory of Molecular Oncology, Harbin, Heilongjiang, China; ^7^ Heilongjiang Cancer Institute, Harbin, Heilongjiang, China

**Keywords:** colorectal cancer, deep learning, whole slide image, pathology, prognosis

## Abstract

**Introduction:**

The conventional tumor-node-metastasis (TNM) classification system remains limited in accurately forecasting prognosis and guiding adjuvant chemotherapy decisions for patients with colorectal cancer (CRC). To address this gap, we introduced and validated a novel pathomics signature (PS_CRC_) derived from hematoxylin and eosin-stained whole slide images, leveraging a deep learning framework.

**Methods:**

This retrospective study analyzed 883 slides from two independent cohorts. An interpretable multi-instance learning model was developed to construct PS_CRC_, with SHapley Additive exPlanations (SHAP) and gradient-weighted class activation mapping (Grad-CAM) for the improvement of model interpretability and the identification of critical histopathological features, respectively. The transcriptomic data was provided by The Cancer Genome Atlas (TCGA) and integrated to investigate the biological mechanisms underpinning PS_CRC_.

**Results:**

The results demonstrated that PS_CRC_ was proven to be an independent prognostic indicator for both overall and disease-free survival. It significantly enhanced the prognostic performance alongside TNM staging, as shown by improvements in net reclassification and integrated discrimination indices. Furthermore, patients in stages II and III with low PS_CRC_ levels were more likely to benefit from chemotherapy. Morphologically, PS_CRC_ reflected features such as tumor infiltration, adipocyte presence, fibrotic stroma, and immune cell engagement. Transcriptome analysis further revealed links between PS_CRC_ and pathways involved in tumor progression and immune evasion.

**Discussion:**

Our findings suggested that the application of deep learning to histopathological images could be an efficient method to improve the prognostic accuracy and evaluate the treatment responses in CRC. The PS_CRC_ offers a promising aid for clinical decision-making by shedding light on key pathogenic processes. Nevertheless, further validation through prospective studies remains essential.

## 1 Introduction

Globally ranked the third in terms of diagnostic frequency, colorectal cancer (CRC) remains the second major cause of cancer mortality (1). Current clinical management predominantly depends on the tumor-node-metastasis (TNM) classification system (2). Nonetheless, notable variability in patient outcomes persists even among individuals categorized within the same clinical stage (3). This outcome disparity underscores the limitations of the TNM system alone and highlights the urgent need for more refined and individualized prognostic biomarkers.

Although recent advances in molecular omics have uncovered critical biomarkers linked to CRC prognosis and progression (4), their translation into routine clinical practice has been hindered by issues such as sample integrity, processing time, and financial burden. In addition, individual driver mutations and RNA-based signatures have demonstrated limited prognostic value and insufficient utility in informing treatment decisions (5, 6). Consequently, there is an ongoing need for novel robust biomarkers that can classify patients into clinically meaningful subgroups, enabling personalized therapeutic approaches, enhancing clinical decision-making, and reducing the risk of inappropriate treatment intensity (7).

The integration of whole-slide imaging and artificial intelligence (AI) has recently transformed the analysis of hematoxylin and eosin (H&E)-stained tissues, a standard yet pivotal step in solid tumor diagnosis. This advancement has enabled more widespread and quantitative evaluation of pathological features. High-resolution digital slides capture rich biomedical information that remains largely untapped, yet hold potential for inferring molecular profiles and predicting clinical outcomes (8, 9). Leveraging such data offers a cost-efficient strategy for enhanced risk stratification by using routinely available histopathological slides.

Despite significant progress, several barriers hinder the clinical integration of deep learning-based pathology analysis. A major limitation is the lack of model interpretability, commonly described as the “black-box” dilemma (10, 11). Additionally, the generalizability of these models remains constrained because of their dependence on the size and heterogeneity of training datasets (12). Other persistent issues include overfitting, limited reproducibility, substantial computational demands, and ethical considerations in medical practice (13, 14).

To overcome these limitations, this study applied weakly supervised learning to analyze whole-slide images (WSIs) and establish a novel prognostic marker for patients with primary CRC. In addition, visualization methods were employed to uncover consistent histopathological patterns correlated with clinical outcomes. To further enhance biological interpretability, we integrated transcriptomic data with morphological features using bioinformatic analyses to elucidate the potential pathobiological mechanisms underlying risk stratification produced by the pathomics model.

## 2 Materials and methods

This study received approval from the institutional ethics committee (ID: KY2024-16) and adhered to the Reporting Recommendations for Tumor Marker Prognostic Studies (REMARK) guidelines (15). Written informed consent was obtained from all participants prior to surgery, including permission to use the tissue specimens and clinical data for research purposes. All the adopted procedures involving human participants followed the ethical principles of the Declaration of Helsinki.

### 2.1 Patient cohorts and study design

This retrospective multicenter cohort research included patients experiencing radical resection for CRC, drawing from three independent cohorts: TCGA-COAD, TCGA-READ, and real-world (HMUCH) cohort. The TCGA-COAD and TCGA-READ cohorts were combined to form a unified meta-cohort, designated as TCGA-CRC. The training cohort consisted of 485 consecutive patients treated at HMUCH between January 2012 and December 2013.

Patients were eligible for inclusion if they met the following criteria: (1) histopathologically confirmed CRC with R0 surgical margins; (2) survival of at least 90 days postoperatively to minimize bias from surgical quality (16, 17); (3) no prior history of malignancy; and (4) availability of complete clinical, pathological, and follow-up records. Individuals who received neoadjuvant therapy were excluded, as such treatment may alter tissue morphology in H&E-stained slides and affect the prognostic assessment.

A total of 398 CRC patients meeting the same eligibility criteria were obtained from TCGA database through the National Cancer Institute’s Genomic Data Commons (https://gdc.cancer.gov/). These cases, which included complete prognostic data and high-quality digital H&E-stained histopathological images, served as the validation cohort.

Baseline clinical and pathological characteristics were comprehensively collected, including patient age, sex, tumor site, invasion depth, perineural invasion, lymphovascular invasion, vascular invasion, lymph node involvement, TNM stage, follow-up information (duration and survival status), and receipt of postoperative adjuvant chemotherapy.

The determination of follow-up duration was performed from the surgery date to the most recent follow-up, with survival status documented at the final visit. Overall survival (OS) was set as the interval between either last follow-up or death and surgery. Disease-free survival (DFS) refers to the time from surgery to the first occurrence of recurrence at any site or death from any cause, whichever occurs earlier.

### 2.2 Image acquisition and data preprocessing

Slides from the HMUCH-CRC cohort were prepared through routine histopathological processing involving fixation in 4% neutral formaldehyde, paraffin embedding, 4 μm sectioning, and H&E staining. TNM staging was subsequently reassessed according to the 8th edition criteria of the American Joint Committee on Cancer (AJCC). For each case, representative sections illustrating the invasion depth were carefully selected. Following quality control, the slides were scanned using an Aperio AT2 scanner (Leica Biosystems, Germany) at 20× optical magnification (0.5 μm/pixel). The resulting digital images were stored in SVS format and managed using Aperio ImageScope software (version 12.4.6).

To facilitate the processing of WSIs approaching 10 gigapixels in size, we first applied the OTSU thresholding algorithm to remove white background regions (18). Subsequently, we partitioned the non-background region into non-overlapping image patches measuring 512 × 512 pixels at a 20-fold optical magnification and recorded their respective locations, resulting in over 7.7 million patches. Note that the batch size is 32, the initial learning rate is 0.01, the cosine decay optimizer is SGD and the momentum is 0.9. Additionally, we applied the Macenko method to normalize the color of small tiles (19), followed by z-score normalization on RGB channels to achieve a standard normal distribution of image intensities.

To enhance model generalization, various data augmentation strategies, such as flipping, mirroring, blurring, mild color perturbations, and progressive sprinkling, were randomly applied to the images in the validation and training sets. Notably, no augmentation was conducted on test images.

### 2.3 Pathomics feature extraction from images

In this study, we designed an advanced deep learning framework to address the complexity and heterogeneity inherent in large-scale tumor histopathology images. The model adopts a two-stage architecture, beginning with patch-level inference and subsequently integrating patch probabilities through a multi-instance learning (MIL)-based feature fusion algorithm to generate WSI-level predictions.

During training, each image patch was assigned the same label corresponding to the patient’s 5-year survival status. For patch-level classification, we employed ResNet-18, an established convolutional neural network architecture renowned for its success in the ImageNet challenge, to estimate patch-level survival likelihoods. Model optimization was performed using softmax cross-entropy loss and mini-batch stochastic gradient descent (SGD).

To enhance generalizability across heterogeneous cohorts, we applied transfer learning by initializing model weights using pre-trained parameters from the ImageNet dataset. The learning rate was fine-tuned via a cosine annealing schedule, defined as


$$\eta_{t}=\eta_{min}^{i}+\frac{1}{2}\left(\eta_{max}^{i}-\eta_{min}^{i}\right)(1+cos(\frac{T_{cur}}{T_{i}}\pi ))$$

$\eta_{min}^{i}=0$ indicates the minimum learning rate, and $\eta_{max}^{i}=0.01$ represents the maximum learning rate. The term $T_{i}=30$ denotes the number of iteration epochs used in the model training. We also utilize transfer learning algorithms to ensure optimal model fitting, by fine-tuning the backbone component parameters when $T_{cur}=\frac{1}{2}T_{i}$ . The learning rate for the backbone component is defined as follows:


$$\eta_{t}^{backbone}=\{\begin{array}{c} \;\;\;\;\;\;\;\;\;\;0\;\;\;\;\;\;\;\;\;\;\;\;\;\;\;\;\;\;\;\;\;\;\;\;\;\;\;\;\;\;\;\;\;\;if\;\;T_{cur}\leq \frac{1}{2}T_{i}\\ \eta_{min}^{i}+\frac{1}{2}(\eta_{max}^{i}-\eta_{min}^{i})(1+cos(\frac{T_{cur}}{T_{i}}\pi ))\;\;\;if\;\;T_{cur}>\frac{1}{2}T_{i} \end{array}\;$$

Following the model training, each patch was assigned a prediction label along with its corresponding probability. These patch-level likelihoods were then aggregated using a classifier to generate the WSI-level outcomes. To facilitate this process, we developed two distinct MIL pipelines: the Patch Likelihood Histogram (PALHI) method and the Bag of Words (BoW) method, inspired by histogram-based and vocabulary-based strategies, respectively.

In the PALHI pipeline, a histogram-based representation is used to quantify the distribution of patch-level likelihoods within each WSI. In contrast, the BoW approach encodes each patch as a floating-point value using term frequency–inverse document frequency (TF-IDF) with a feature vector representing the entire slide.

Using these two distinct pipelines, patch-level outputs were effectively transformed into WSI-level features. Each method contributed 101 probabilistic features and 2 categorical label features. These individual feature sets were then integrated through early fusion, resulting in a unified feature vector of 206 dimensions for subsequent analysis.

### 2.4 Construction of the pathomics signature

The least absolute shrinkage and selection operator (LASSO) Cox regression model that incorporates an L1 penalty for the toward-zero reduction of feature coefficients is a well-established method for survival analysis in high-dimensional settings (20, 21). Referred to as the tuning constant, the penalty parameter λ governs the penalty strength.

In this study, the 10-fold cross-validation with the minimum criteria was applied for obtaining the optimal λ by minimizing the partial likelihood deviance within the training set. This approach enabled the identification of key prognostic features and construction of a formula for calculating the pathomics signature. The application of the derived formula to the validation set helped compute the corresponding signature scores.

### 2.5 Prognostic value of the pathomics signature

Through using the maximally selected rank statistics within the training cohort, the identification of the optimal cutoff for the pathomics signature was achieved, which was subsequently tested within the validation set. This threshold was adopted for the classification of the patients into high- and low-signature categories for prognostic evaluation. The differences in OS and DFS between both categories were analyzed using restricted mean survival time (RMST) metrics and Kaplan-Meier (K-M) survival curves (22).

The independent prognostic significance of the pathomics signature was assessed through performing univariate and multivariate Cox regression analyses. Subgroup heterogeneity was examined using interaction-based subgroup analysis. To evaluate the potential influence of unmeasured confounding, E-value analysis was conducted as a sensitivity assessment (23).

To measure discriminative performance, we calculated the concordance index (C-index) and the area under the receiver operating characteristic curve (AUROC). The agreement between predicted and observed survival probabilities was evaluated through applying calibration plots. The clinical utility of the pathomics model was further examined using decision curve analysis (DCA), which quantifies net benefit across varying decision thresholds (24).

To determine the added value of the pathomics signature beyond conventional TNM staging, we evaluated its impact on discrimination, calibration, clinical benefit, integrated discrimination improvement (IDI), net reclassification improvement (NRI), and prediction error curves (25).

### 2.6 Interpretation of the pathomics signature

To mitigate the interpretability limitations of deep learning models, we applied SHapley Additive exPlanations (SHAP), a method rooted in cooperative game theory, to quantify the contribution and relative importance of individual features to model outputs (26). This technique enables both global and instance-level interpretation of the predictions generated by the trained model.

The gradient-weighted class activation mapping (Grad-CAM) was utilized to produce heatmaps over selected image tiles for further exploration of prognostically relevant morphological patterns (27), highlighting crucial regions that influenced network predictions. This visualization technique utilized gradient information from the last convolutional layer of our deep learning network, providing a visual explanation that facilitates understanding and validating of the model’s decision-making process.

### 2.7 Bioinformatics analyses of the pathomics signature

Transcriptomic profiles from TCGA cohort were retrieved with the TCGAbiolinks package (28). Gene set enrichment analysis (GSEA) was performed to infer the biological processes related to the pathomics signature (29). Additionally, pathway activity was quantified using gene set variation analysis (GSVA) via the GSVA package (30), allowing the identification of significantly enriched pathways across different patient subgroups. Functional interpretation was based on the well-curated “hallmark gene sets” (31).

Weighted correlation network analysis (WGCNA) was performed using the WGCNA package, which aims to identify the pathomics signature-related gene modules (32). The scale-free topology fitting index of 0.85 was set as the threshold to construct the signed weighted gene co-expression network. The minimum co-expression module size was set to 30, and the merge cut minimum module merge cut height was set to 0.25. A biweight midcorrelation coefficient (bicor) > 0.1 and *P-value* < 0.05 were selected as the thresholds to find gene modules significantly associated with the pathomics signature. Gene annotation enrichment analysis was performed using the clusterProfiler package (33).

Furthermore, according to the guideline for transcriptome-based cell-type quantification methods, we utilized the MCPcounter and xCell algorithms to quantify the proportions of specific immune and stromal cells within the CRC samples (34–36).

### 2.8 Statistical analysis

The comparison of the continuous variables with normal distributions were performed using unpaired two-sample t-tests, while the analysis of the non-normally distributed variables were achieved via the Mann-Whitney U test or Kruskal-Wallis test. The assessment of the categorical variables was achieved via either Fisher’s exact test or the chi-squared (χ^2^) test. Survival curves were generated through applying the K-M method and evaluated via the log-rank test. Univariate and multivariate associations were examined using Cox regression analysis with 95% confidence intervals (CIs) and hazard ratios (HRs). The associations between continuous variables were analyzed through the calculation of the Spearman rank correlation coefficients. All statistical analyses were conducted using R software (v4.0.5) and SPSS (v19.0). Deep learning experiments were implemented in Python (v3.7.12). All tests were two-sided, with *P-value* < 0.05 considered statistically significant.

## 3 Results

### 3.1 Clinicopathological characteristics

Detailed clinicopathological features of patients from the training cohort (n = 485) and the validation cohort (n = 398) are summarized in **Supplementary Table S1**. Across all 883 patients, the median age was 62 years with the interquartile range (IQR) of 54–71, where males accounted for 54.9% (485/883) of the population. The majority (86.2%, 761/883) was diagnosed at stage II or III. In the training set, the median follow-up period was 72.5 months (IQR: 56.47–121.23), with 5-year DFS and OS rates of 75.51% and 81.28%, respectively. In contrast, the validation cohort had a shorter median follow-up of 24.33 months (IQR: 15.24–36.53), with corresponding 5-year DFS and OS rates of 61.89% and 70.97%. The differences observed in the clinicopathological profiles between cohorts reflect real-world clinical diversity, thereby enhancing the generalizability of our results.

### 3.2 Pathomics signature construction

**Figure 1:**
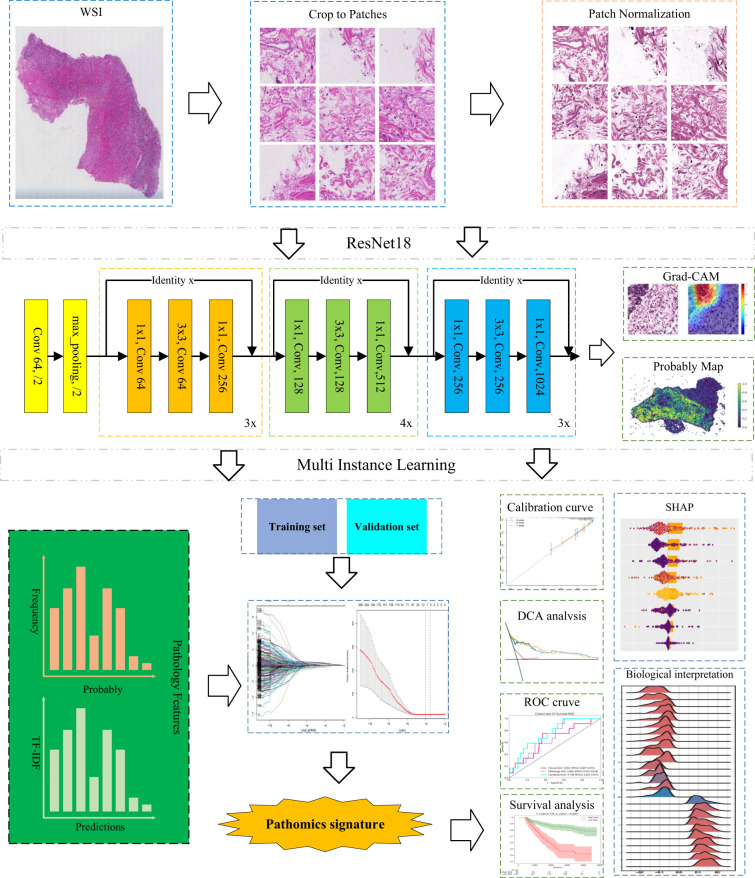
Construction framework of the pathomics signature.


The development framework for the pathomics signature is depicted in **Figure 1**. In the training cohort, a LASSO-Cox regression model with 10-fold cross-validation was employed to construct the signature. Using the optimal penalty parameter λ (**Supplementary Figure S1**), eight selected pathomics features were integrated into a composite risk score. The final formula for calculating the pathomics signature is as follows:

$$\begin{array}{c} \text{Pathomics}\;\text{signature}\\ =0.464259187\times HistogramBoWProb\_0.15+0.516838374\\ \times HistogramBoWProb\_0.66+0.76054024\\ \times HistogramBoWProb\_0.72+0.000727042\\ \times BoWProb\_008-0.379252147\times BoWProb\_06\\ -0.475519653\times BoWProb\_063-0.090370453\\ \times BoWProb\_068+0.050898359\times BoWPred\_0 \end{array}$$

The optimal cutoff point, identified based on the maximum standardized log-rank statistic, was 0.1139008. Patients in both the training and validation cohorts were stratified into high- and low-signature groups accordingly. Associations between the pathomics signature and clinicopathological characteristics are presented in **Supplementary Table S2**. Notably, a potential correlation was observed between the signature and lymph node counts.

### 3.3 Prognostic value of the pathomics signature

**Figure 2:**
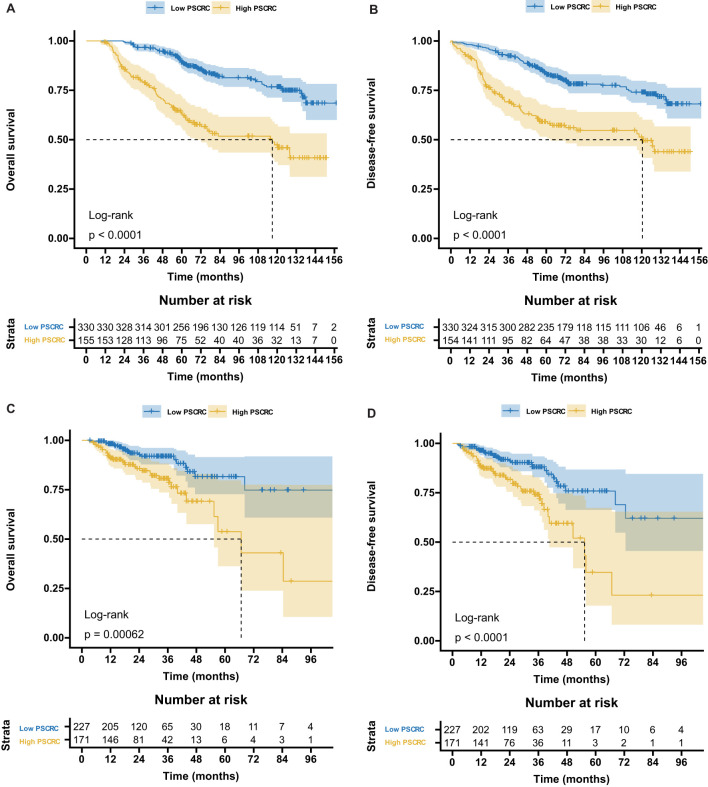
Kaplan-Meier survival curves according to the pathomics signature. **(A)** The OS rate difference between the high- and low- PS_CRC_ patients in the training cohort. **(B)** The DFS rate difference between the high- and low- PS_CRC_ patients in the training cohort. **(C)** The OS rate difference between the high- and low- PS_CRC_ patients in the validation cohort. **(D)** The DFS rate difference between the high- and low- PS_CRC_ patients in the validation cohort; OS, overall survival; DFS, disease-free survival; PS_CRC_, pathomics signature of colorectal cancer.

**Supplementary Figure S2** illustrates the distribution of pathomics signature values by survival status along with selected feature profiles, indicating a positive association between elevated signature scores and a higher risk of recurrence or mortality. K-M survival analysis (**Figure 2**) demonstrated significant differences in both OS and DFS between the low- and high-signature groups in the training and validation cohorts.

RMST analysis revealed a sustained survival advantage for patients with low pathomics signature scores across multiple time points, with the magnitude of the benefit increasing over time (**Table 1**). Specifically, the low-signature group exhibited an OS advantage of approximately 2 months at year 3, 8 months at year 5, and a notable 15 months by year 7 when compared to the high-signature group.

**Table 1:** Restricted mean survival time (RMST) difference analyses in the training and validation cohorts.

Time point	High PScrc			Low PScrc			RMST difference^#^			*P* value
	RMST	95% CI		RMST	95% CI		Effect size	95%CI		
Training cohort (n = 485)										
OS										
2 years	23.196	22.821	23.571	23.997	23.992	24.002	-0.801	-1.176	-0.427	**<0.0001**
3 years	33.052	32.032	34.073	35.734	35.57	35.897	-2.681	-3.715	-1.648	**<0.0001**
5 years	50.092	47.585	52.6	58.407	57.734	59.079	-8.314	-10.911	-5.718	**<0.0001**
7 years	63.754	59.612	67.896	78.913	77.475	80.351	-15.159	-19.543	-10.774	**<0.0001**
9 years	76.173	70.186	82.16	98.394	95.958	100.831	-22.221	-28.685	-15.757	**<0.0001**
DFS										
2 years	21.632	20.769	22.495	23.541	23.25	23.832	-1.909	-2.82	-0.999	**<0.0001**
3 years	30.345	28.774	31.915	34.824	34.287	35.362	-4.48	-6.14	-2.82	**<0.0001**
5 years	45.619	42.437	48.8	56.114	54.909	57.318	-10.495	-13.897	-7.093	**<0.0001**
7 years	59.176	54.253	64.1	75.346	73.273	77.42	-16.17	-21.512	-10.828	**<0.0001**
9 years	72.303	65.501	79.105	94.002	90.895	97.109	-21.699	-29.177	-14.221	**<0.0001**
Validation cohort (n = 398)										
OS										
2 years	22.317	21.598	23.035	23.398	23.049	23.746	-1.081	-1.879	-0.282	**0.008**
3 years	32.245	30.859	33.631	34.431	33.634	35.227	-2.186	-3.784	-0.588	**0.0074**
5 years	48.735	45.231	52.239	54.738	52.502	56.974	-6.003	-10.16	-1.847	**0.0046**
7 years	59.781	52.06	67.501	73.245	68.559	77.931	-13.464	-22.496	-4.433	**0.0035**
9 years	66.717	54.053	79.381	91.192	83.142	99.242	-24.475	-39.481	-9.469	**0.0014**
DFS										
2 years	21.74	20.911	22.569	23.05	22.57	23.53	-1.309	-2.267	-0.351	**0.0074**
3 years	30.988	29.423	32.554	33.824	32.877	34.771	-2.836	-4.665	-1.006	**0.0024**
5 years	44.217	40.326	48.108	52.867	50.344	55.391	-8.651	-13.288	-4.013	**0.0003**
7 years	50.548	42.671	58.425	69.194	64.038	74.35	-18.646	-28.061	-9.231	**0.0001**
9 years	56.097	43.008	69.186	84.089	74.872	93.305	-27.992	-43.999	-11.984	**0.0006**

OS, overall survival; DFS, disease-free survival; PS_CRC_, pathomics signature of colorectal cancer; RMST, restricted mean survival time; CI, confidence interval; #, RMST difference = RMST_high-pathomics signature_ - RMST_low-pathomics signature_.

Univariate and multivariate Cox regression analyses confirmed the pathomics signature as an independent predictor of both DFS and OS in the training cohort (**Table 2**). Consistent findings were observed in the validation cohort (**Supplementary Table S3**). To assess the robustness of these associations against potential unmeasured confounding, E-value sensitivity analyses were conducted based on adjusted HRs in both cohorts (**Supplementary Table S4**).

**Table 2:** Univariate and multivariate Cox regression analyses of the pathomics signature and clinicopathological characteristics for overall survival and disease-free survival in the training cohort.

Variables	Samples	Overall survival				Disease-free survival			
		Univariate analysis		Multivariate analysis		Univariate analysis		Multivariate analysis	
		HR (95% CI)	*P* value	HR (95% CI)	*P* value	HR (95% CI)	*P* value	HR (95% CI)	*P* value
**Age, years**	485	1.036 (1.020, 1.053)	**<0.0001**	1.035 (1.019, 1.052)	**<0.0001**	1.032 (1.017, 1.048)	**<0.0001**	1.027 (1.012, 1.044)	**0.0006**
Sex									
Male	274	Reference				Reference			
Female	211	0.724 (0.509, 1.030)	0.073			0.792 (0.566, 1.109)	0.174		
Tumor location									
Left side	213	Reference				Reference			
Right side	212	0.893 (0.618, 1.290)	0.547			0.863 (0.605, 1.230)	0.414		
Rectum	60	1.285 (0.765, 2.159)	0.343			1.180 (0.714, 1.951)	0.518		
VELI									
No	440	Reference				Reference			
Yes	45	1.863 (1.117, 3.107)	**0.0171**	1.132 (0.656, 1.952)	0.656	2.047 (1.273, 3.293)	**0.0031**	1.451 (0.875, 2.406)	0.149
Perineural invasion									
No	398	Reference				Reference			
Yes	87	1.735 (1.165, 2.584)	**0.0067**	1.422 (0.929, 2.177)	0.105	1.972 (1.359, 2.859)	**0.00034**	1.583 (1.067, 2.350)	**0.023**
Lymph node harvest									
≤ 12	106	Reference				Reference			
> 12	379	0.878 (0.590, 1.307)	0.521			0.727 (0.503, 1.050)	0.089		
Depth of invasion									
T1-2	16	Reference				Reference			
T3	265	1.326 (0.417, 4.223)	0.633			2.286 (0.560, 9.330)	0.249		
T4	204	1.713 (0.538, 5.456)	0.362			3.160 (0.775, 12.885)	0.109		
Lymph node metastasis									
N0	329	Reference				Reference			
N1	117	1.983 (1.354, 2.905)	**0.0004**	1.892 (1.278, 2.803)	**0.0015**	1.736 (1.196, 2.519)	**0.0037**	1.506 (1.023, 2.216)	**0.038**
N2	39	2.492 (1.446, 4.293)	**0.001**	2.033 (1.157, 3.572)	**0.014**	2.593 (1.551, 4.336)	**0.00028**	2.029 (1.185, 3.474)	**0.01**
Distant metastasis									
M0	481	Reference				Reference			
M1	4	6.144 (1.939, 19.461)	**0.002**	1.943 (0.529, 7.129)	0.317	5.560 (1.758, 17.587)	**0.0035**	1.692 (0.486, 5.891)	0.409
MSI status									
MSI-H	17	Reference				Reference			
MSS	60	1.203 (0.255, 5.666)	0.815			2.799 (0.354, 22.100)	0.329		
**Pathomics signature**	485	3.598 (2.919, 4.435)	**<0.0001**	3.475 (2.785, 4.336)	**<0.0001**	2.378 (2.024, 2.793)	**<0.0001**	2.235 (1.886, 2.650)	**<0.0001**

VELI, venous emboli and/or lymphatic invasion; MSI, microsatellite instable; MSS, microsatellite stable; HR, hazard ratio; CI, confidence interval.The bold value means the P < 0.05.

Stratified analyses based on clinicopathological variables demonstrated that the pathomics signature remained a significant prognostic marker across all subgroups, except for patients with perineural invasion in the validation cohort (**Table 3**). A potential interaction between age and lymph node harvest was suggested by the subgroup difference testing. No other significant interaction effects were observed, thereby supporting the overall robustness of the pathomics signature as a prognostic factor.

**Table 3:** Subgroup analysis for the pathomics signature among different clinical features in the training and validation cohorts.

Variables	Training cohort (n = 485)					Validation cohort (n = 398)				
	Samples	Overall survival		Disease-free survival		Samples	Overall survival		Disease-free survival	
		HR (95% CI)	*P* value for interaction	HR (95% CI)	*P* value for interaction		HR (95% CI)	*P* value for interaction	HR (95% CI)	*P* value for interaction
**Elderly**			**0.0001**		**0.0043**			**0.0493**		0.1311
No (age ≤ 65)	340	4.771 (3.556, 6.403)		2.545 (2.068, 3.130)		181	9.035 (2.699, 30.246)		4.978 (2.151, 11.516)	
Yes (age > 65)	145	2.537 (1.831, 3.514)		2.185 (1.628, 2.934)		217	2.931 (2.070, 4.149)		2.470 (1.814, 3.364)	
**Early-onset**			0.1959		**0.0058**			0.1774		0.1817
No (age ≥ 50)	380	3.431 (2.735, 4.304)		2.238 (1.881, 2.663)		346	3.264 (2.360, 4.514)		2.699 (2.039, 3.572)	
Yes (age < 50)	105	4.822 (3.039, 7.653)		4.623 (2.919, 7.322)		52	13.388 (1.619, 110.704)		8.442 (1.591, 44.786)	
**Sex**					0.6993			0.0643		0.1467
Male	274	3.900 (2.924, 5.202)	0.8968	2.253 (1.847, 2.748)		211	2.759 (1.776, 4.284)		2.310 (1.582, 3.372)	
Female	211	3.114 (2.264, 4.282)		2.477 (1.886, 3.253)		187	5.077 (2.793, 9.227)		3.781 (2.348, 6.089)	
**Tumor type**										
Colon cancer	425	3.757 (3.004, 4.699)	0.3512	2.408 (2.037, 2.847)	0.7078	242	3.499 (2.433, 5.032)	0.9829	2.755 (2.009, 3.776)	0.7906
Rectal cancer	60	2.935 (1.766, 4.879)		2.207 (1.413, 3.448)		156	3.470 (1.791, 6.726)		2.994 (1.747, 5.132)	
**Tumor location**			0.6628		0.9222			0.5554		0.2733
Left side	213	3.804 (2.878, 5.028)		2.425 (1.960, 3.000)		104	7.335 (2.383, 22.574)		6.496 (2.536, 16.639)	
Right side	212	3.202 (2.196, 4.670)		2.120 (1.598, 2.813)		138	4.837 (2.311, 10.122)		2.651 (1.695, 4.146)	
Rectum	60	2.759 (1.571, 4.844)		2.945 (1.702, 5.096)		156	3.157 (1.670, 5.968)		2.892 (1.669, 5.011)	
**VELI**			0.8869		0.368			0.2667		0.4625
No	440	3.581 (2.835, 4.524)		2.335 (1.962, 2.779)		247	4.673 (2.844, 7.676)		3.073 (2.211, 4.270)	
Yes	45	3.574 (2.121, 6.023)		3.154 (1.814, 5.483)		151	2.461 (1.233, 4.915)		2.252 (1.207, 4.203)	
**Perineural invasion**			0.5998		0.857			0.3829		0.2557
No	398	3.276 (2.466, 4.353)		2.249 (1.774, 2.851)		352	3.545 (2.546, 4.936)		2.909 (2.203, 3.841)	
Yes	87	3.367 (2.396, 4.732)		2.498 (1.905, 3.275)		46	1.500 (0.193, 11.639)		1.037 (0.153, 7.045)	
**Lymph node harvest**			0.1059		**0.0108**			0.1467		0.134
≤ 12	106	3.060 (1.805, 5.187)		1.786 (1.316, 2.424)		43	6.527 (1.717, 24.811)		4.907 (1.652, 14.581)	
> 12	379	3.885 (3.066, 4.922)		2.752 (2.260, 3.351)		355	3.324 (2.314, 4.774)		2.669 (1.964, 3.628)	
**Lymph node metastasis**			0.409		0.2053			0.8108		0.6741
N0	329	3.498 (2.649, 4.618)		2.289 (1.858, 2.819)		242	3.339 (2.154, 5.176)		2.572 (1.849, 3.579)	
N+	156	4.389 (2.958, 6.511)		2.860 (2.124, 3.851)		156	3.686 (1.947, 6.977)		3.158 (1.727, 5.778)	
**TNM stage**			0.4018		0.2009			0.9192		0.694
Early (Stage I and II)	328	3.495 (2.647, 4.614)		2.288 (1.858, 2.818)		239	3.300 (2.107, 5.169)		2.534 (1.809, 3.548)	
Advance (Stage III and IV)	157	4.403 (2.966, 6.536)		2.866 (2.127, 3.860)		159	3.625 (1.962, 6.697)		3.143 (1.755, 5.631)	

TNM, tumor-node-metastasis; VELI, venous emboli and/or lymphatic invasion; MSI, microsatellite instable; MSS, microsatellite stable; HR, hazard ratio; CI, confidence interval; #, *P* value for interaction analysis.The bold value means the P < 0.05.

Time-dependent receiver operating characteristic (ROC) curves demonstrated that the pathomics signature achieved favorable predictive performance for 3-, 5-, and 7-year OS and DFS in both the training and validation cohorts (**Supplementary Figure S3**). The corresponding calibration plots further confirmed a strong agreement between the predicted and observed survival probabilities across the same time intervals (**Supplementary Figure S4**).

Moreover, decision curve analysis (DCA) showed that incorporating the pathomics signature into prognostic assessment yielded greater net clinical benefit than either the “treat-all” or “treat-none” strategies in both cohorts (**Supplementary Figure S5**), supporting its potential for real-world clinical application.

### 3.4 Incremental value of the pathomics signature added to the TNM stage

The combined model, which were based on the combination of the pathomics signature and the TNM staging system, exhibited a significantly higher C-Index than the TNM stage, and these results could also be found in the validation cohort (**Supplementary Table S5**).

Furthermore, the AUROCs of the 3 models also confirmed the superior discrimination ability of the combined models for estimating DFS and OS in the training and validation cohorts (**Supplementary Figure S6**). Additionally, compared with the TNM stage models, the combined models were the most accurate models (**Supplementary Figure S7**) and showed greater net benefits across most of the range of reasonable threshold probabilities (**Supplementary Figure S8**).

Finally, the combined model showed a significant NRI and IDI for prognosis estimation compared with the TNM stage model (**Supplementary Table S6**), indicating that the pathomics signature could provide additional prognostic value to the TNM staging system for CRC.

### 3.5 Pathomics signature and benefits of adjuvant chemotherapy

To evaluate the predictive utility of the pathomics signature in the context of adjuvant chemotherapy, we analyzed its association with survival outcomes in stage II and III CRC patients stratified by postoperative adjuvant chemotherapy status. In both the training and validation cohorts, adjuvant chemotherapy significantly improved OS and DFS in these subgroups (**Supplementary Figure S9**). Furthermore, the pathomics signature demonstrated a significant correlation with OS and DFS, regardless of whether the patients received adjuvant therapy (**Supplementary Figure S10**).

**Figure 3:**
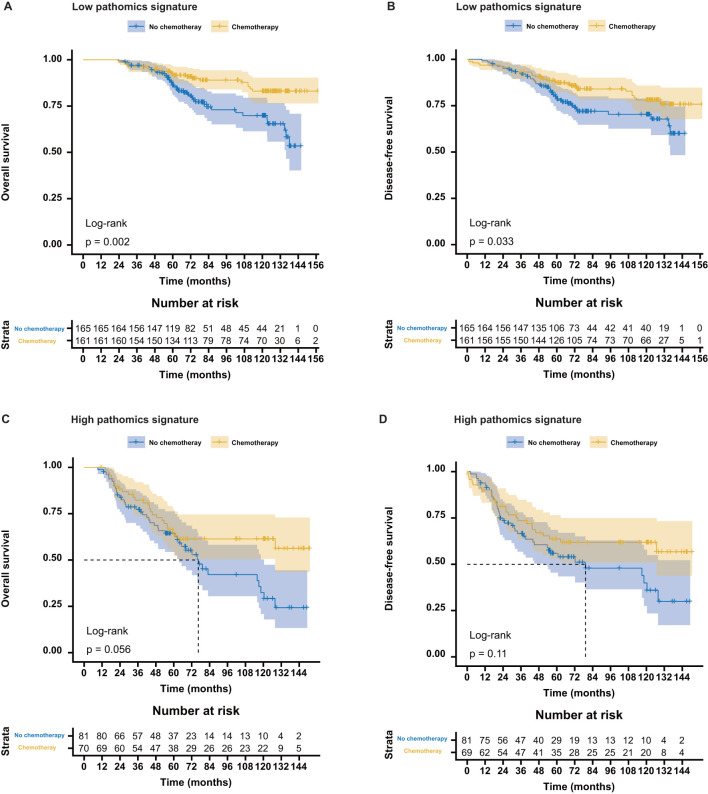
Association between the pathomics signature and survival benefits from adjuvant chemotherapy in stage II and stage III colorectal cancer. **(A, B)** Survival benefits from adjuvant chemotherapy for the low- PS_CRC_ patients. **(C, D)** Survival benefits from adjuvant chemotherapy for the high-PS_CRC_ patients. PS_CRC_, pathomics signature of colorectal cancer.

Among the patients in the low-pathomics signature group, adjuvant chemotherapy was significantly associated with improved OS and DFS. In contrast, this survival benefit was not observed in the high-pathomics signature group (**Figure 3**). Further interaction analysis revealed a significant effect modification, indicating that individuals with low pathomics signature scores derived greater benefits from adjuvant chemotherapy than those with high scores (**Supplementary Table S7**).

### 3.6 Interpretation of the pathomics signature

**Figure 4:**
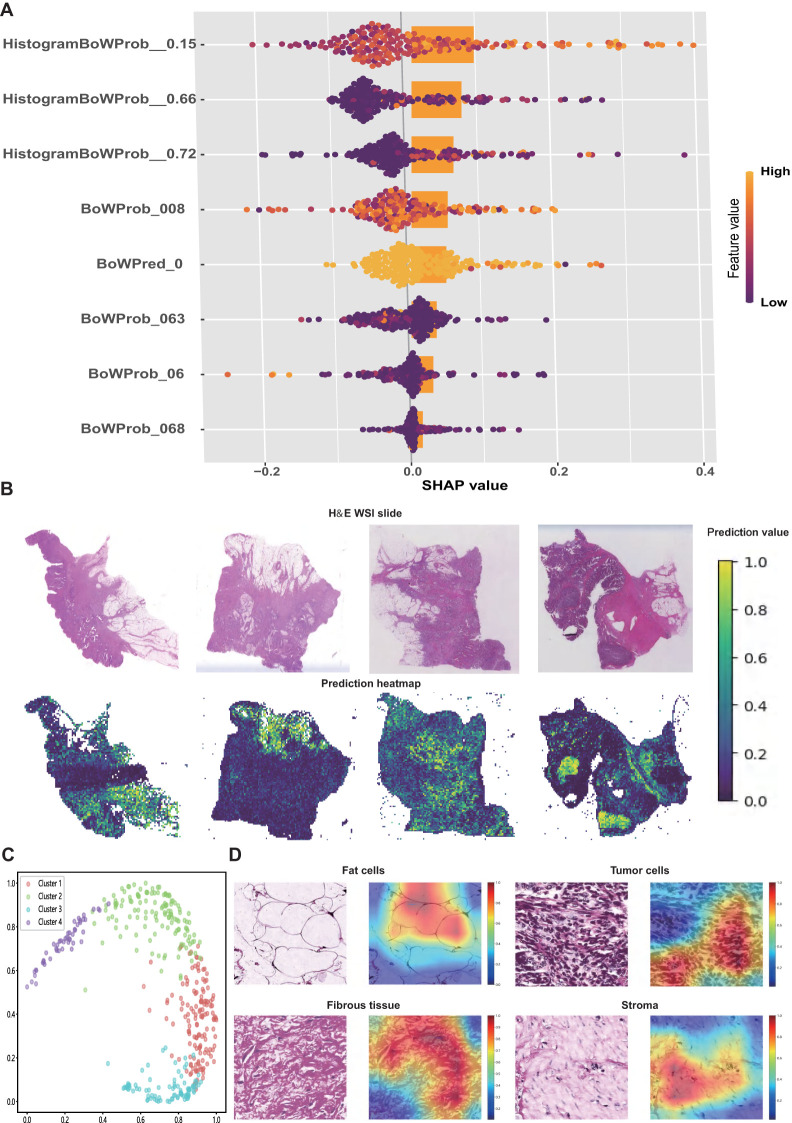
Interpretation of the pathomics signature. **(A)** SHAP values for the individual features of pathomics signature. **(B)** Representative H&E slide and their predicted heatmaps. **(C)** The four potential features extracted from heatmaps were separated by the random tree algorithm. **(D)** Visualisation of the heatmaps of high-risk features related to the pathomics signature.

SHAP values were used to interpret the contribution of individual features to the model predictions. As illustrated in the SHAP summary plot (**Figure 4A**), *HistogramBoWProb_0.15* emerged as the most influential feature, closely followed by *HistogramBoWProb_0.66*, *HistogramBoWProb_0.72*, and *BoWProb_008*. In contrast, *BoWProb_068* contributed the least among the eight pathomics features.

To further interpret the spatial features linked to patient prognosis, prediction heatmaps were generated to highlight the key regions contributing to the model output (**Figure 4B**). High-risk cases are typically marked by dense tumor stroma, abundant tumor cells, and muscle tissue infiltration. In contrast, low-risk regions were predominantly characterized by normal mucosa, loose stroma, and inflammatory infiltration.

To further elucidate the model’s decision-making process, Grad-CAM was applied to extract informative visual cues. The top 500 most influential regions were selected to explore the dominant histopathological patterns associated with patient survival. By clustering the highest-ranked image patches, four distinct histological clusters were identified using a random tree algorithm (**Figure 4C**).

Subsequently, expert pathologists reviewed and annotated the representative regions identified using the model. Key histological components, including tumor cells, adipocytes, fibrous tissue, and stroma, were highlighted in red (**Figure 4D**). These features appeared to be closely associated with an elevated risk of recurrence and mortality, offering a morphological interpretation of the predictive elements underlying the pathomics signature.

### 3.7 Association between the pathomics signature and biological features

**Figure 5:**
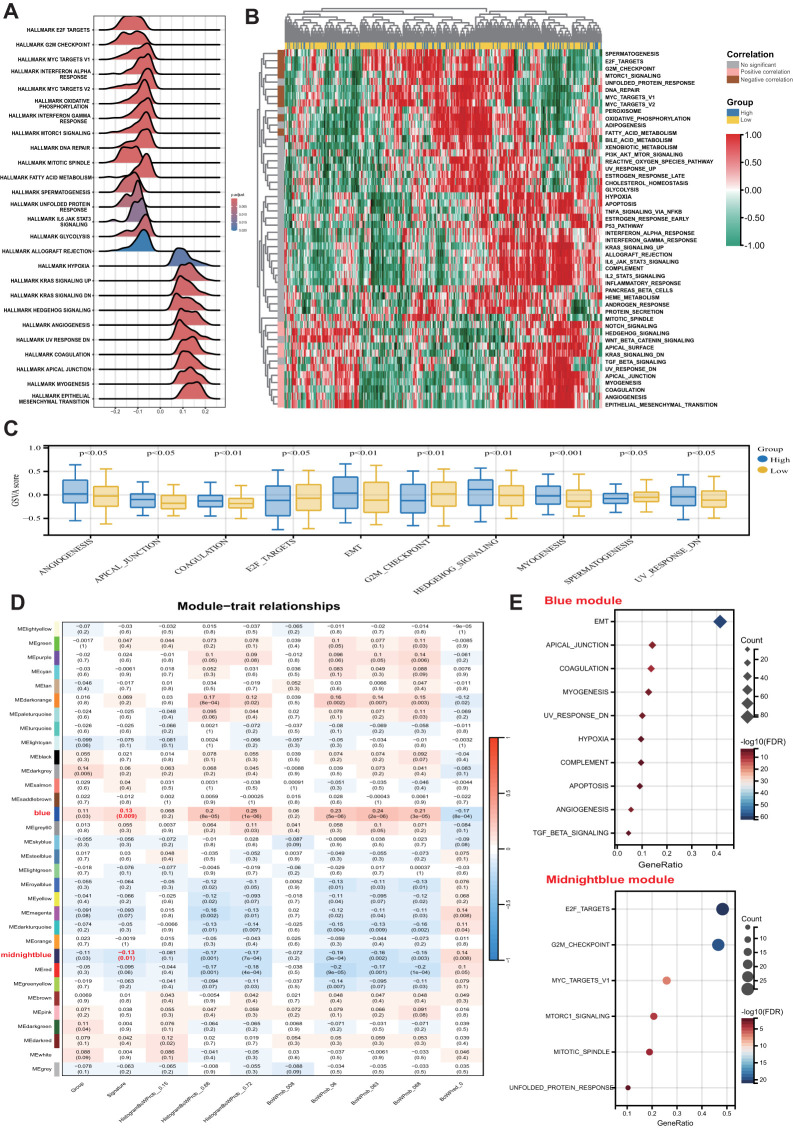
Biological features of the pathomics signature. **(A)** GSEA of the hallmark gene sets for the pathomics signature. **(B)** Heatmap shows GSVA enrichment scores of the hallmark gene sets. **(C)** The bar plot shows the different analysis outcomes for GSVA scores of hallmark gene sets between the high- and low-pathomics signature groups. **(D)** Module-trait relationships. Each row shows a module eigengene; each column corresponds to a clinical trait. Each cell contains the corresponding correlation (upper number) and p-value (lower number). **(E)** Functional enrichment analysis of the hallmark gene sets for genes in the blue and midnightblue modules. GSEA, gene set enrichment analysis; GSVA, gene set variation analysis.

GSEA was initially conducted to investigate potential biological mechanisms associated with the pathomics signature (**Figure 5A**). CRC samples with low pathomics signature scores exhibited significant enrichment in pathways related to DNA repair, proliferation, metabolism, and immune functions. Conversely, samples with high pathomics signature scores showed the activation of canonical oncogenic signaling and invasion-related pathways.

GSVA further confirmed the significant functional differences between the high- and low-pathomics signature groups (**Figures 5B, C**). Angiogenesis, epithelial-mesenchymal transition (EMT), and other invasion-associated pathways were significantly upregulated in the high-pathomics signature group. In contrast, pathways such as spermatogenesis, E2F targets, and G2M checkpoint were more active in the low-pathomics signature group.

To identify gene co-expression patterns linked to the pathomics signature, WGCNA was performed using the top 5,000 most variable genes defined by the median absolute deviation (MAD). A cluster dendrogram was generated with an optimal soft threshold power of 14 (**Supplementary Figure S11A**), resulting in 32 distinct colored modules (**Supplementary Figure S11B**). Unassigned genes were grouped into the grey module and excluded from further analysis. Correlation analysis between module eigengenes and the pathomics signature identified two modules, blue and midnight blue, as significantly associated (*|bicor|* > 0.1 and *P-value* < 0.05) (**Figure 5D**). Within these modules, gene significance was strongly correlated with module membership (**Supplementary Figure S11C**), suggesting that these genes may play pivotal roles in shaping essential biological roles related to the pathomics signature.

Subsequent functional enrichment analysis of these modules revealed distinct biological profiles (**Figure 5E**). Genes in the midnight blue module were predominantly enriched in proliferation-related pathways, whereas genes in the blue module were associated with invasion, metastasis, and immune-related processes. These findings indicate that the pathomics signature accurately reflects the underlying biological features associated with the multiple crucial hallmarks of CRC.

### 3.8 Association between the pathomics signature and tumor microenvironment

**Figure 6:**
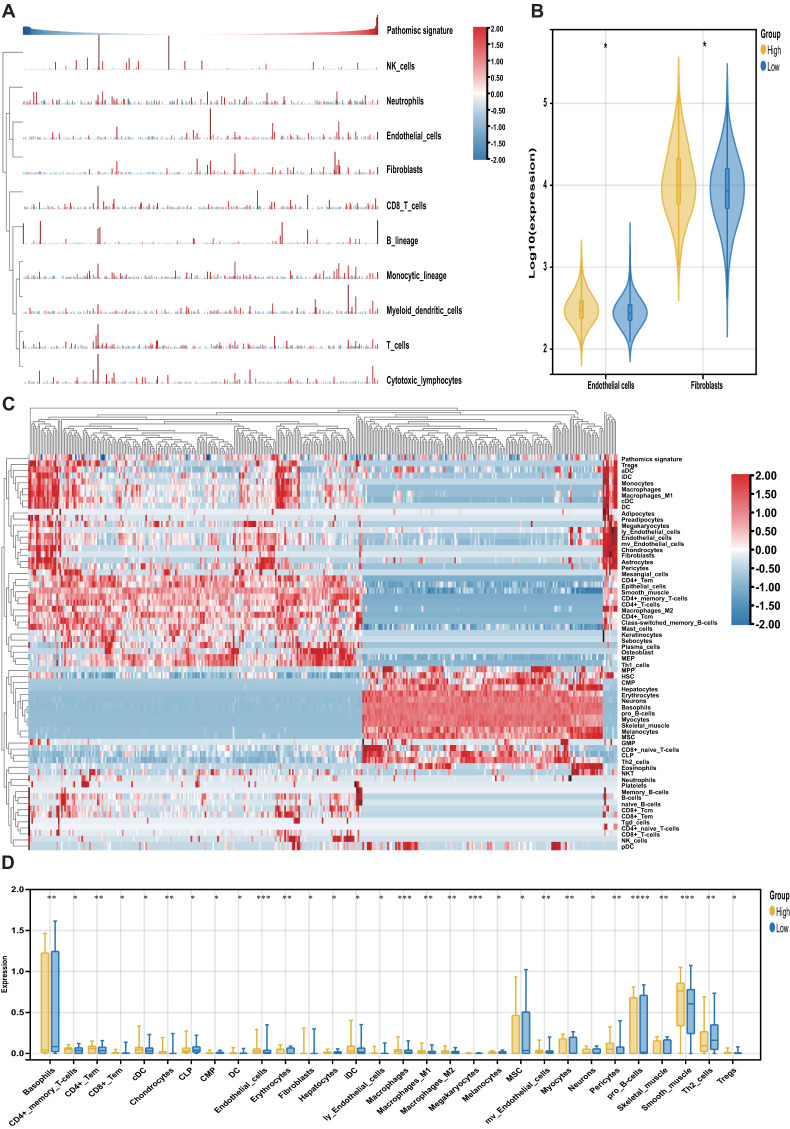
Association of the pathomics signature and tumor microenvironment. **(A)** Heatmap shows the infiltration level of stromal and immune cells derived from the MCPcounter algorithm in relation to the pathomics signature. **(B)** Different analyses for the MCPcounter-derived cells between the different subgroups. **(C)** Heatmap shows the infiltration level of stromal and immune cells derived from the xCell algorithm in relation to the pathomics signature. **(D)** Different analyses for the xCellr-derived cells between the different subgroups. P values were obtained by the Wilcoxon test. The asterisks represented the statistical P-value (*P < 0.05; **P < 0.01; ***P < 0.001; ****P < 0.0001).

The MCPcounter algorithm was applied to estimate the relative abundance of stromal and immune cell subsets in relation to the pathomics signature. Both fibroblasts and endothelial cells were positively correlated with the pathomics signature and were significantly enriched in the high-pathomics signature group (**Figures 6A, B**).

To further characterize tumor microenvironment heterogeneity, the xCell algorithm was used (**Figure 6C**). Consistent with MCPcounter results, a positive correlation was observed between the pathomics signature and various endothelial and stromal cell types. Moreover, a higher proportion of lymphoid and myeloid cells was detected in samples with elevated pathomics scores. In contrast, several stem, stromal, and lymphoid cells exhibited negative associations with the pathomics signature, underscoring their relevance in reflecting CRC tumor microenvironment (**Figure 6D**).

Finally, we explored the prognostic relevance of these pathomics signature-related cells in CRC (**Supplementary Table S8**). Survival analysis showed that cell types positively correlated with the pathomics signature were linked to poorer outcomes, whereas those negatively correlated were associated with favorable prognosis. These findings suggest that the pathomics signature captures distinct non-tumor cellular components that contribute to differential clinical trajectories in CRC.

## 4 Discussion

Accurate prognostic assessment and identification of adjuvant chemotherapy benefits remain essential for the effective risk stratification and clinical management of CRC. In this study, we developed a pathomics signature comprising eight features derived from digital H&E-stained slides using LASSO-Cox regression modeling. The signature consistently demonstrated strong prognostic value across different follow-up periods and was applicable to both colon and rectal cancer cases. Notably, incorporating the pathomics signature into conventional TNM staging systems significantly improved predictive performance compared to the use of the TNM staging system alone, underscoring its potential as a complementary tool for CRC prognosis.

Adjuvant chemotherapy remains the standard treatment for patients with advanced CRC (7). However, the considerable variability in clinical outcomes among individuals with identical TNM staging and treatment regimens indicates that a significant proportion of patients do not derive meaningful benefits from adjuvant chemotherapy (3). Our findings revealed that patients with low pathomics signature scores were more likely to benefit from adjuvant chemotherapy, whereas those with high scores exhibited limited therapeutic gain. These results suggest that the pathomics signature may serve as a valuable stratification tool to guide personalized treatment decisions and optimize therapeutic efficacy.

Consistent with our results, previous studies have shown that AI-derived pathomics signatures can function as novel prognostic biomarkers for CRC. Some of these approaches rely on handcrafted features extracted from pathologist-annotated regions of interest (ROIs) within WSIs using specialized tools to compute predefined descriptors (20, 37). However, such methods are often time-consuming, prone to subjectivity, and difficult to reproduce (38). Meanwhile, these predefined image features have a limited ability to represent image information.

Recently, an increasing number of studies have employed deep neural network-based approaches to directly predict survival outcomes from histopathological images (39–44). Although deep learning has demonstrated excellent performance in medical image analysis, its “black-box” nature has raised high concerns, which may limit acceptability by clinicians and researchers, and may not be appropriate for high-level decision-making, such as those related to oncological prognosis or predicting treatment benefits (10).

Unlike traditional “black-box” deep learning models, our approach integrates the predictive power of deep learning with the interpretability of the LASSO method, enabling a more physically interpretable model construction that facilitates assessing the significance of each input variable through SHAP values. Additionally, we employed the Grad-CAM technique to visualize the regions that contributed most to our model, aiding in identifying critical morphological features for survival status. Our findings suggest that for patients with poor prognosis, the model is more attentive to the adipose tissue surrounding the tumor, which is in line with previous research (40, 42).

We further explored the transcriptomic associations to uncover the molecular underpinnings of our model. Significant differences in stromal and immune cell infiltration were observed between the high- and low-pathomics signature groups. The high-pathomics signature group demonstrated significantly elevated stromal infiltration, particularly involving endothelial cells and fibroblasts. Such stromal enrichment has been implicated in promoting tumor progression and resistance to therapy, leading to poor prognosis (45). Moreover, this enhanced stroma infiltration suggests that patients in the high-pathomics signature group might exhibit an immune-excluded or immune-desert phenotype and display reduced responsiveness to immunotherapy (46).

Meanwhile, GSEA analyses revealed that differentially expressed genes between two groups were enriched in pathways related to proliferation, metabolism, immune dysregulation, and EMT. This finding suggests that tumors in the high-risk group displayed enhanced invasiveness and metastatic potential. Furthermore, the enrichment results suggest that the cell cycle might play a pivotal role in CRC prognosis, as evidenced by the enriched gene sets associated with E2F targets, MYC targets, and G2M checkpoints. These results highlight the potential of targeting the cell cycle as a therapeutic strategy for the treatment of CRC.

In addition to the advantages in interpretability, in feature mining, the conventional strategy was sampling based on tumor area (20, 39, 42), which may result in the loss of other critical prognostic features present in the tumor microenvironment. Moreover, random single-patch sampling from the entire WSI fails to retain important spatial relationships between patches (47). However, utilizing our MIL deep learning model with a dataset consisting of 7.7 million patches extracted from WSIs of 883 patients, we are able to automatically adjust the contribution of each patch to the overall WSI-level prediction in a learnable manner by assigning higher weights to key patches. This approach not only preserves histological features of tumors and peri-tumoral tissues but also retains spatial information among patches, resulting in improved predictive performance compared to conventional methods.

Despite these promising results, several limitations of this study should be acknowledged. First, its retrospective design introduced potential biases and unmeasured confounders. However, it is unlikely that unmeasured confounding alone could completely explain our findings due to the substantial E-values observed in the main results. We employed rigorous statistical analysis methods to ensure the reliability and interpretability of our findings, offering a foundation for the evolution of algorithmic devices, and facilitating the execution of prospective cohort studies and phase 2 and 3 randomized controlled trials (RCTs). Second, the bioinformatic analyses conducted were based on *post hoc* correlations and do not constitute mechanistic evidence. Thirdly, considering the computational costs, in this study, we adopted a relatively concise model architecture. We utilized only ResNet-18 for patch-level feature extraction and employed the PALHI and BoW methods for WSI-level aggregation. Nonetheless, we still achieved promising results. In recent years, numerous groundbreaking technologies have emerged in the field of pathological image analysis. Recent advances in pathology foundation models and attention-based MIL methods have shown improved performance in feature aggregation. These technologies effectively address the challenges posed by the high-resolution and multi-scale characteristics of pathological images through contrastive learning, graph network optimization, and feature space reshaping, providing new tools for precision medicine. We believe that these complex and advanced network architectures would further optimize the model’s performance. Finally, although the pathomics signature was developed and externally validated using multicenter data from patients across different countries and hospitals, further validation is required to ensure its robustness across diverse populations, sample preparation protocols, and image acquisition platforms encountered in global clinical practice.

Unlike molecular biomarkers, which often require additional testing and incur extra costs, the pathomics signature offers a cost-effective alternative as it is derived from routinely available H&E-stained slides. This approach enables seamless integration into clinical workflows without imposing financial burden. Importantly, the pathomics signature can support more informed decision-making by refining the risk–benefit evaluation of adjuvant chemotherapy, aiding both clinicians and patients in treatment planning.

Based on our findings, for patients with a high pathomics signature, characterized by an unfavorable prognosis and limited benefit from adjuvant chemotherapy, it is crucial to explore alternative treatment strategies such as targeted therapy, immunotherapy, and participation in new clinical trials. Furthermore, rigorous postoperative surveillance is indispensable for promptly identifying any indications of recurrence or metastasis, enabling the timely initiation of appropriate therapeutic interventions.

For patients with a low pathomics signature, it is advisable to consider omitting adjuvant treatment to avoid unnecessary exposure to potentially toxic effects. By sparing these patients from the morbidities and costs associated with adjuvant chemotherapy, it would greatly enhance the current management of CRC. However, further validation in prospective, international, and multicenter randomized trials is warranted to test the clinical utility of the pathomics signature for individualized decision-making. Moreover, current research has confirmed that biomarkers for neoadjuvant chemotherapy can be constructed using deep learning and preoperative biopsy tissue. Given the growing importance of neoadjuvant chemotherapy for individuals with locally advanced CRC, future clinical trials should focus more on this area to investigate the potential clinical value of computational pathology in the management of CRC.

## 5 Conclusion

Our study developed and validated a pathomics signature using MIL deep learning analysis of H&E-stained WSIs to directly predict prognosis for CRC patients. The integration of pathomics signatures can enhance the prognostic value of the TNM staging system and identify patients who may benefit from adjuvant chemotherapy, thereby supporting more informed clinical decision-making. Nevertheless, further verification through prospective studies involving multicenter large patient cohorts is still needed.

## Data Availability

The raw data supporting the conclusions of this article will be made available by the authors, without undue reservation.
