# Supplementary figures and images for: Development and validation of a deep learning-based pathomics signature for prognosis and chemotherapy benefits in colorectal cancer: a retrospective multicenter cohort study
Source: Front Immunol. 2025 Jul 8;16:1602909. doi: 10.3389/fimmu.2025.1602909 (PMC12280904; doi:10.3389/fimmu.2025.1602909)

# A

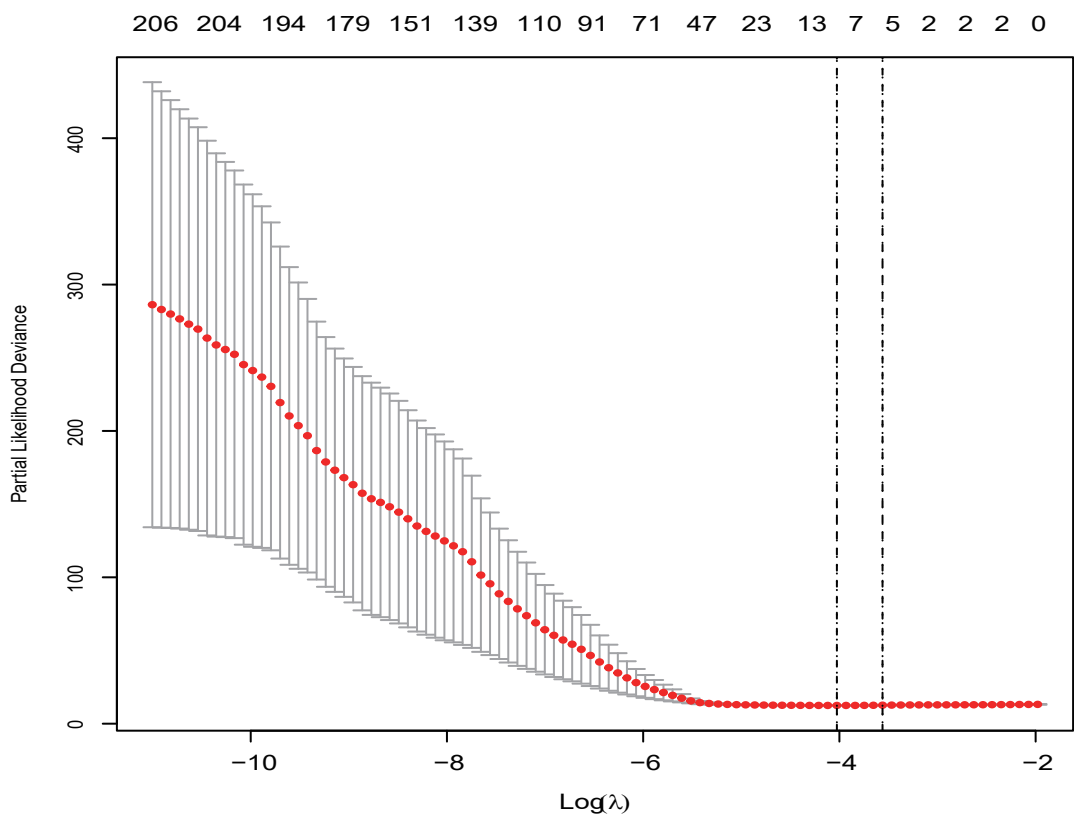

# B

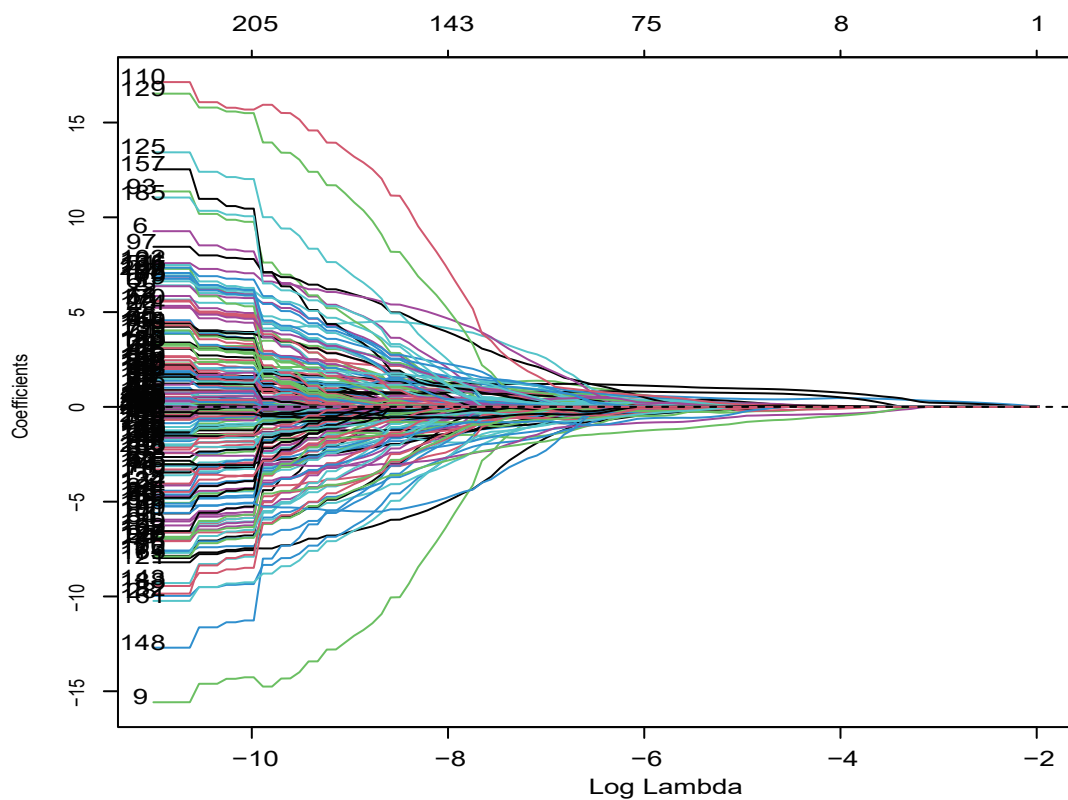

Supplement: Supplementary file 1 [file Image1.pdf]

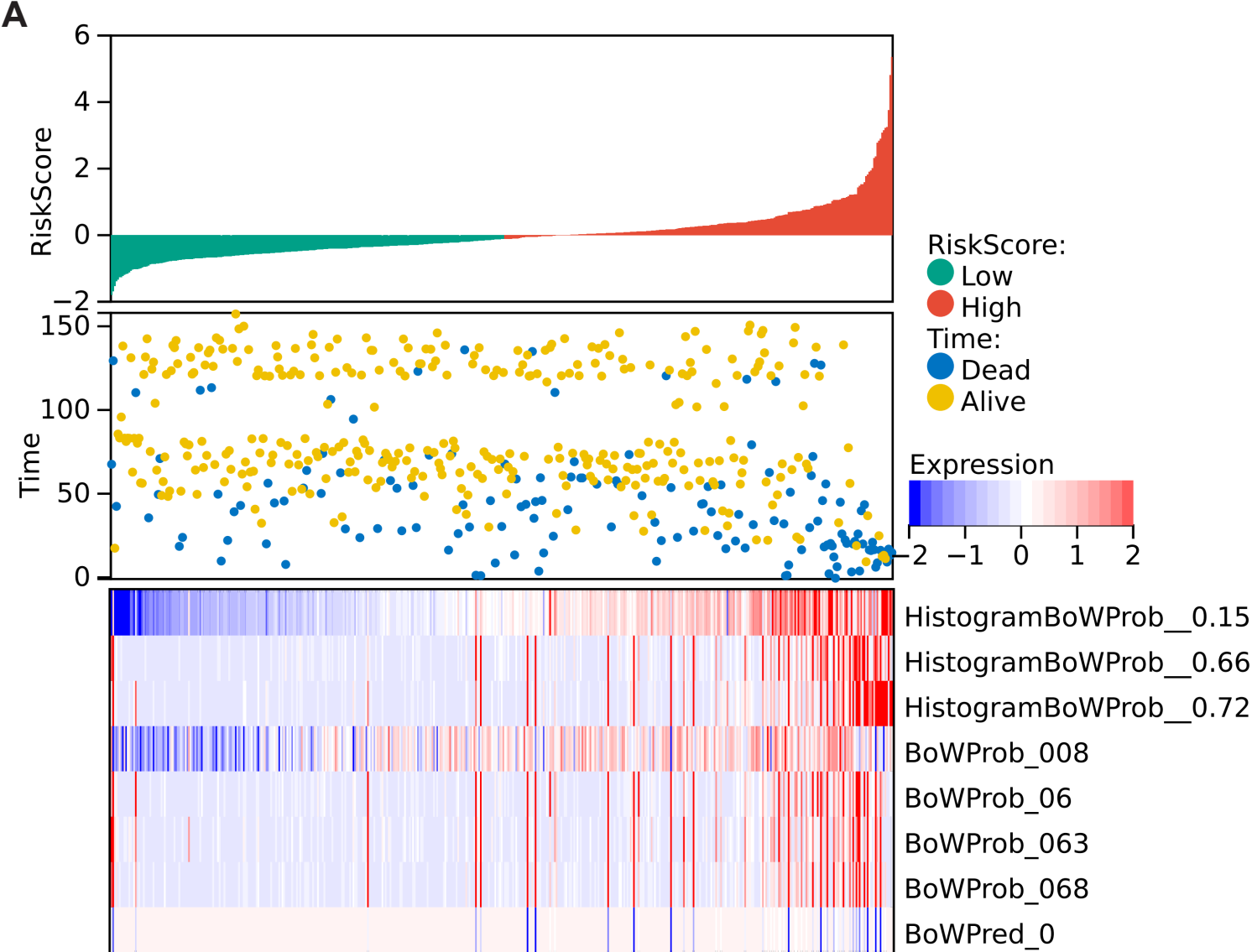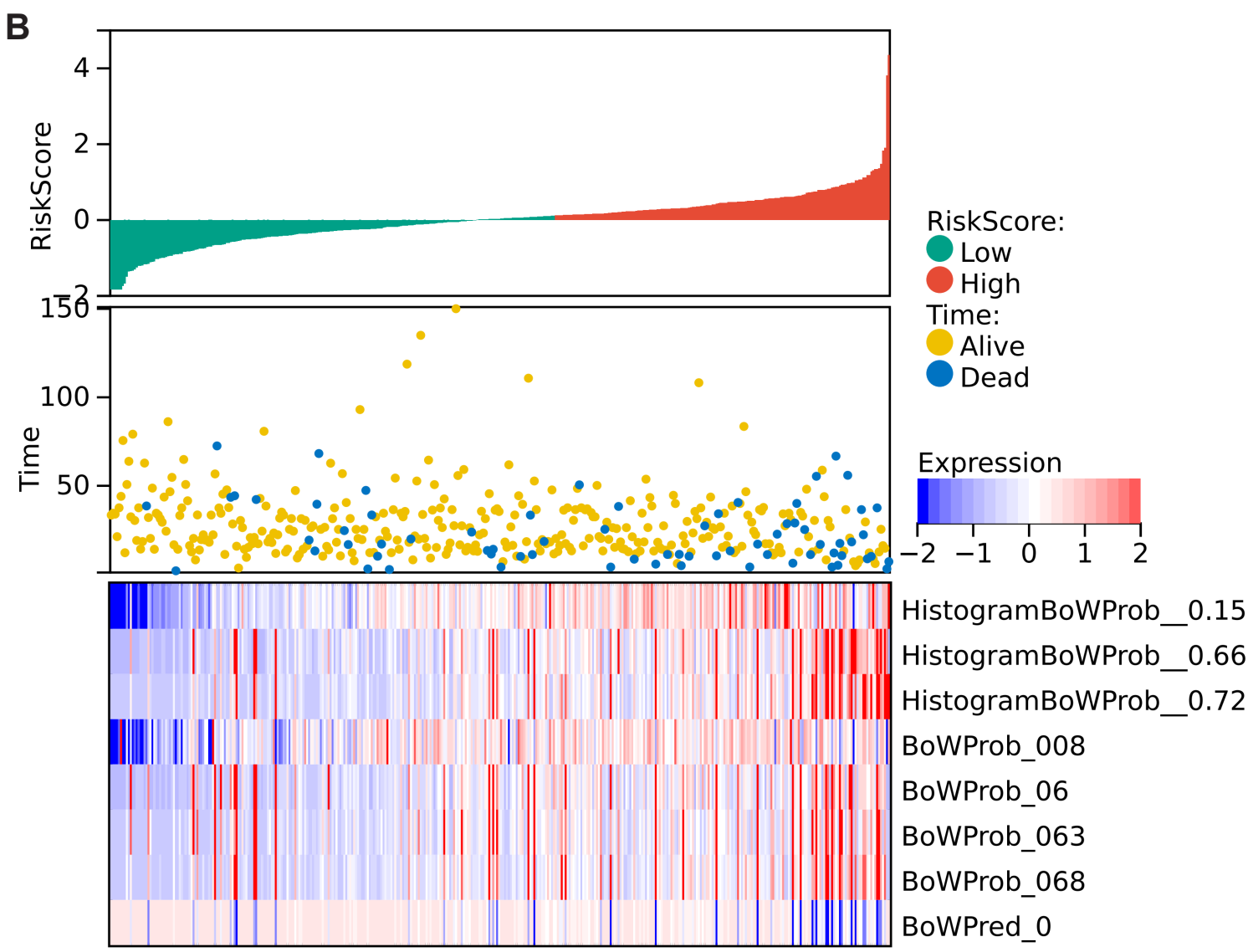

Supplement: Supplementary file 2 [file Image2.pdf]

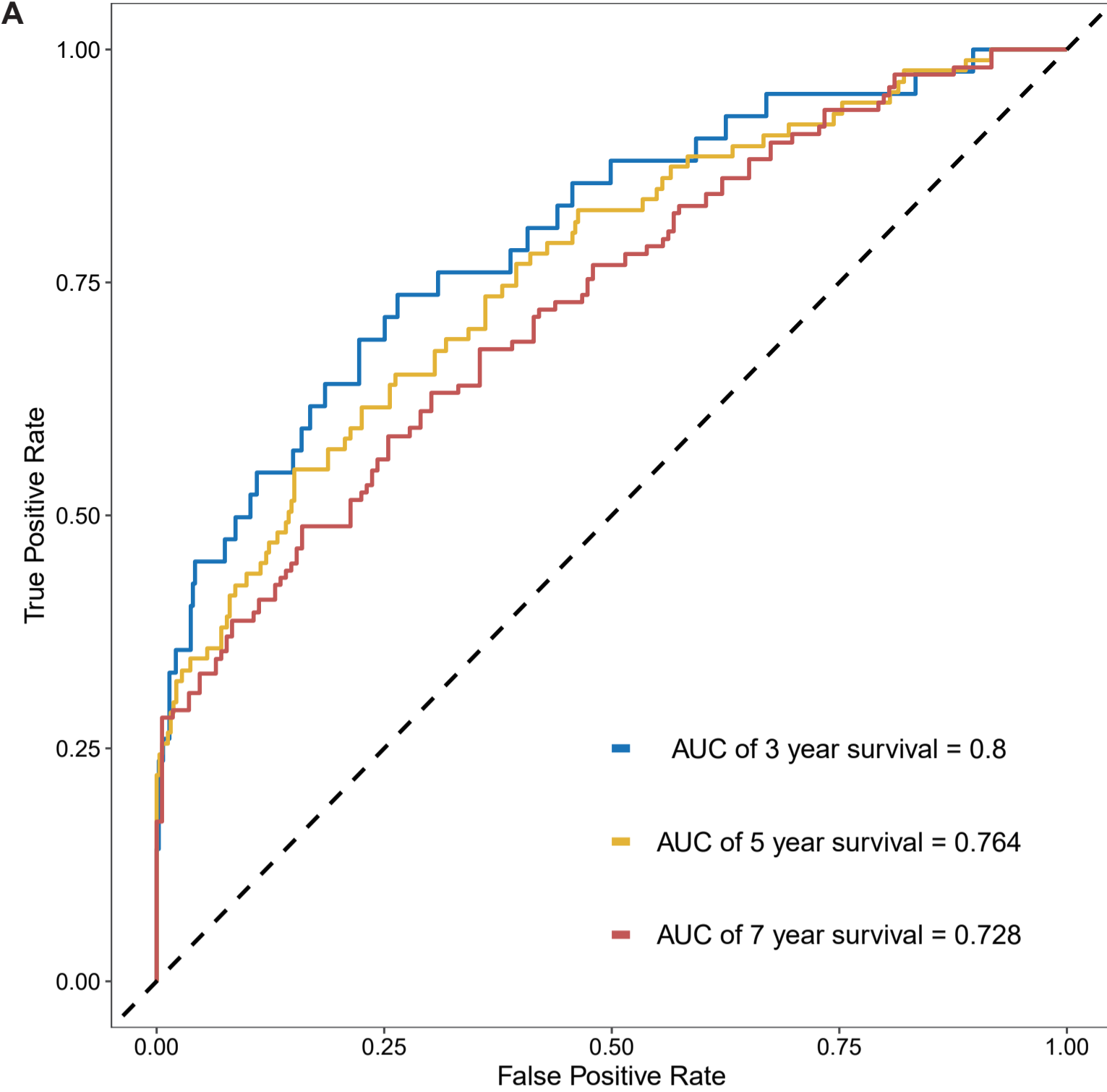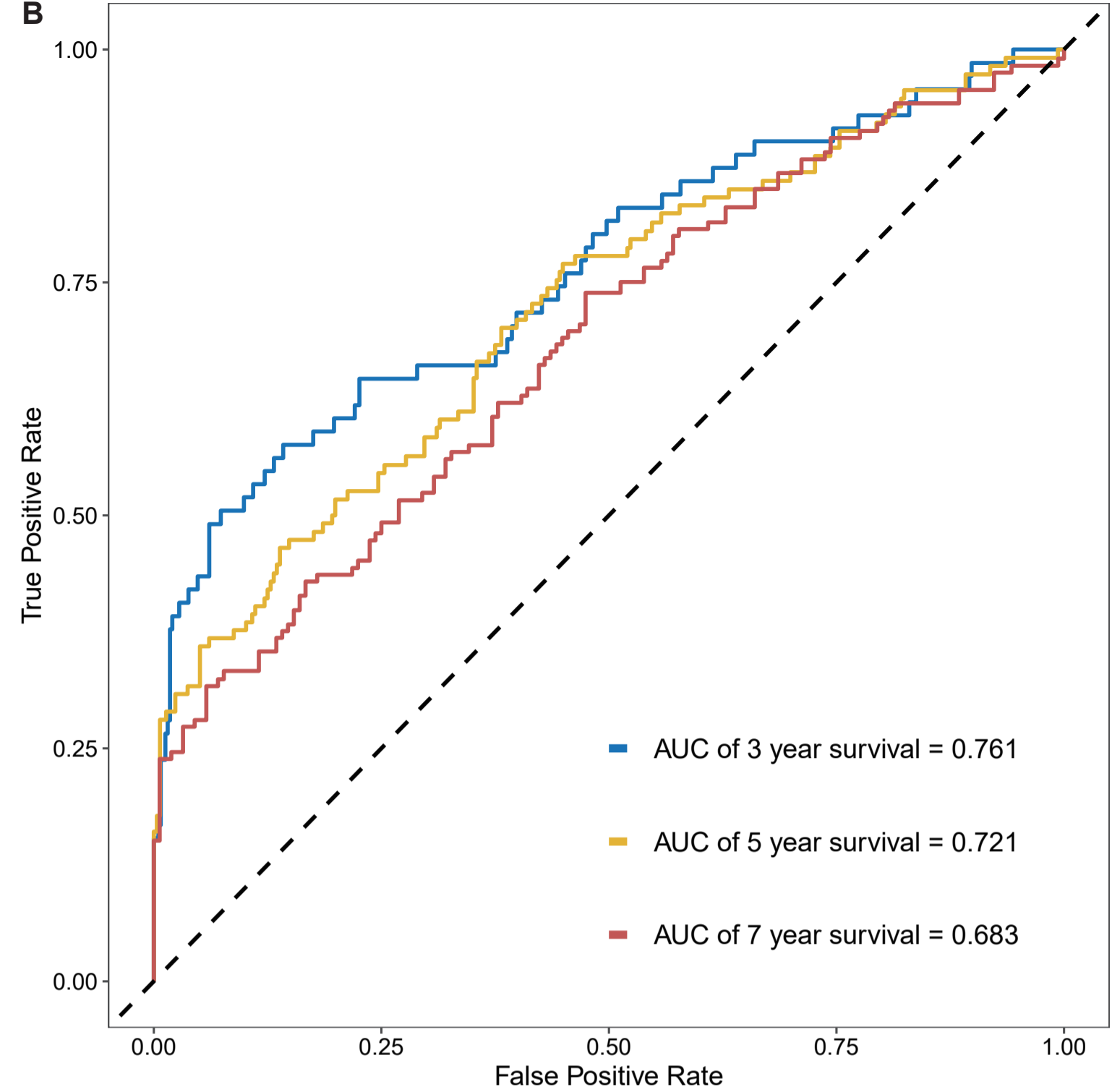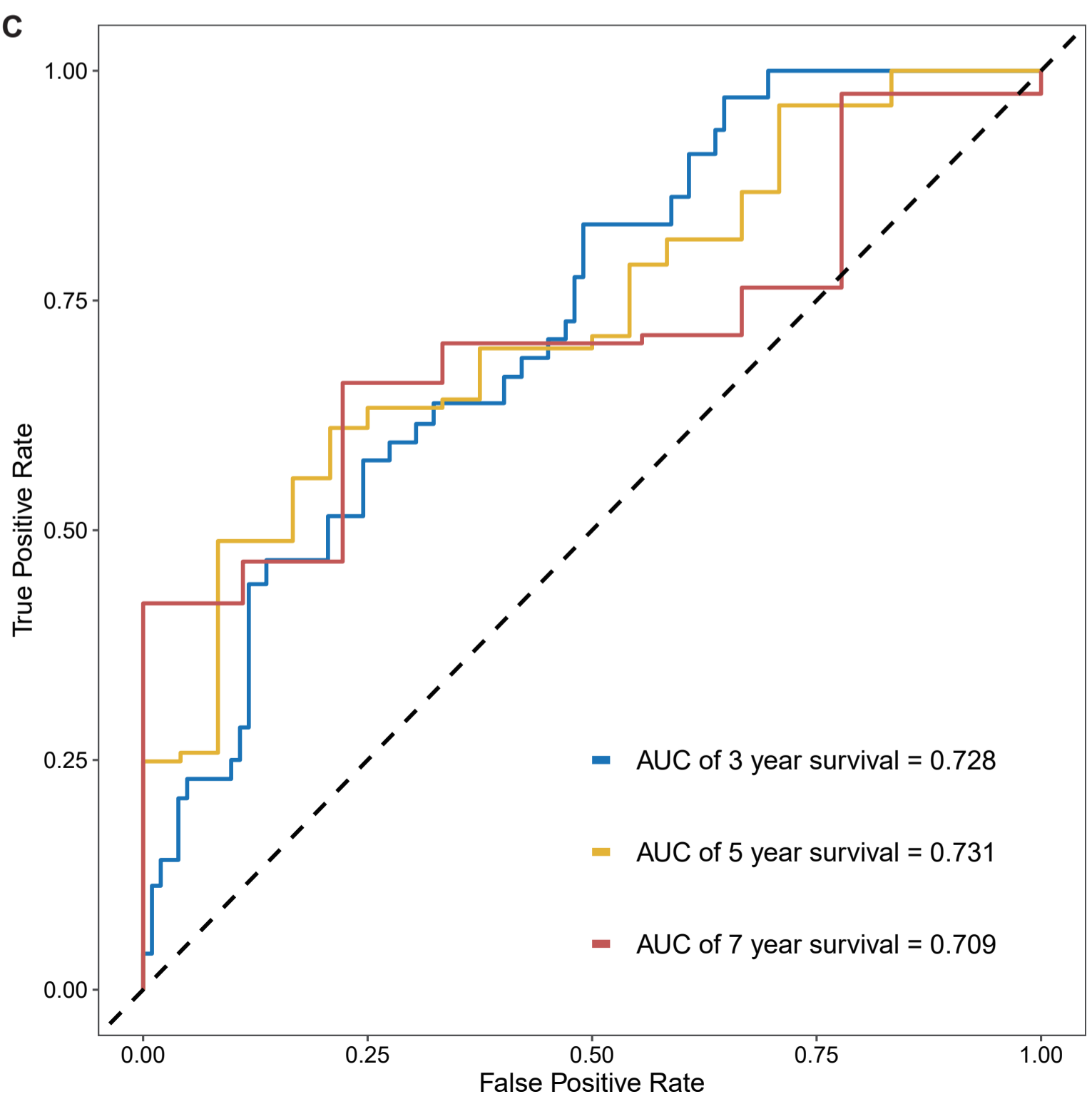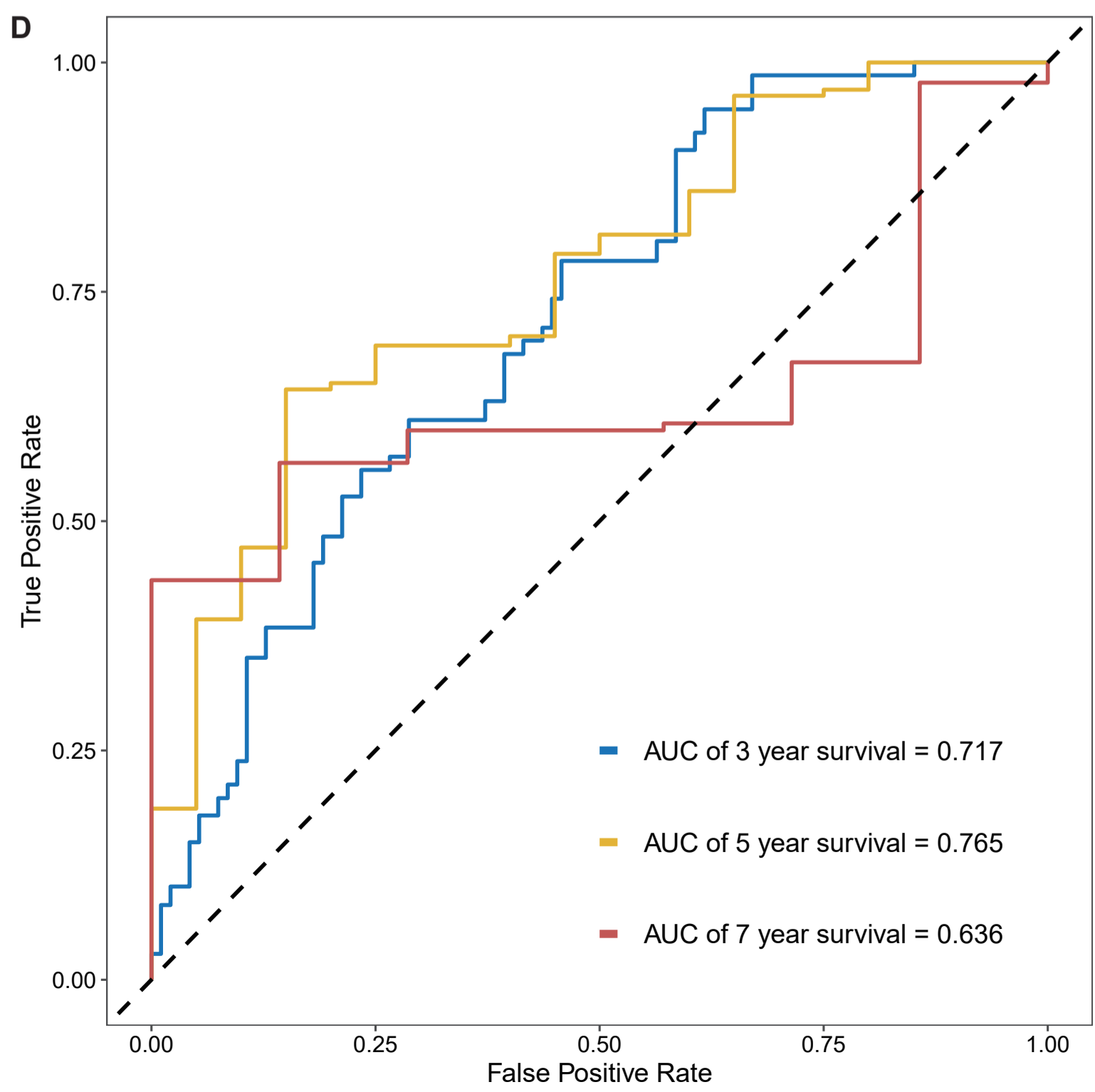

Supplement: Supplementary file 3 [file Image3.pdf]

**A**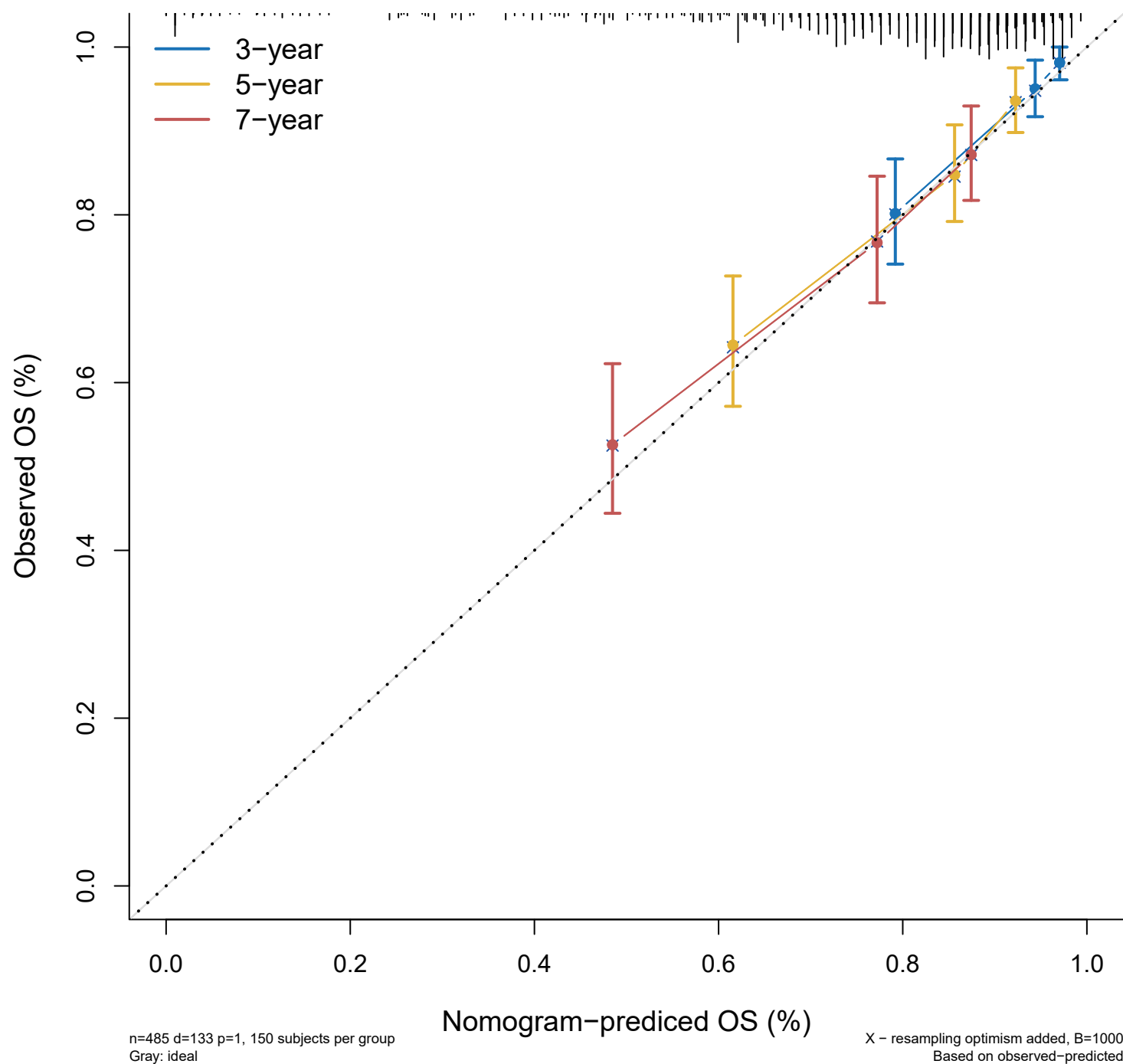**B**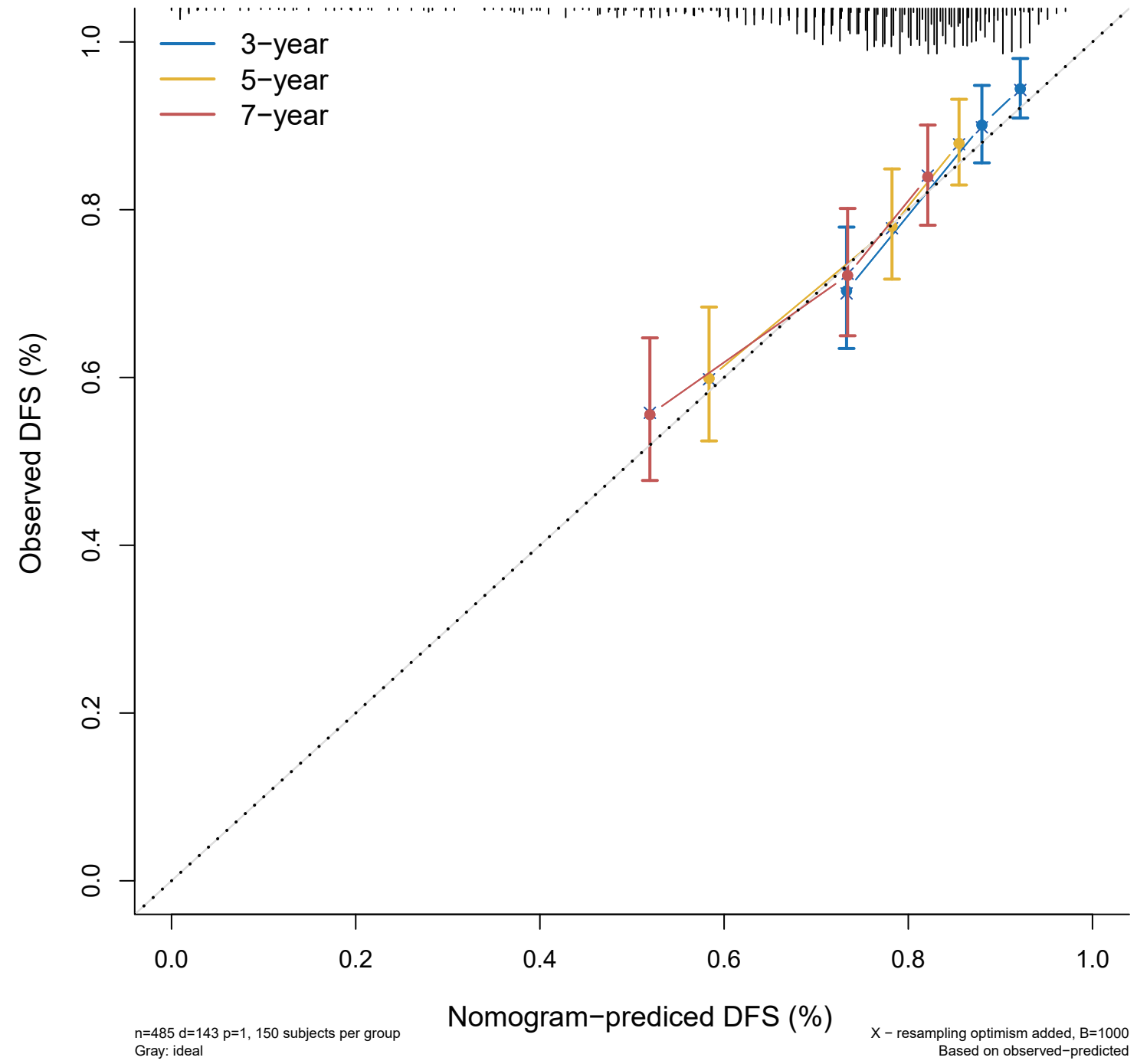**C**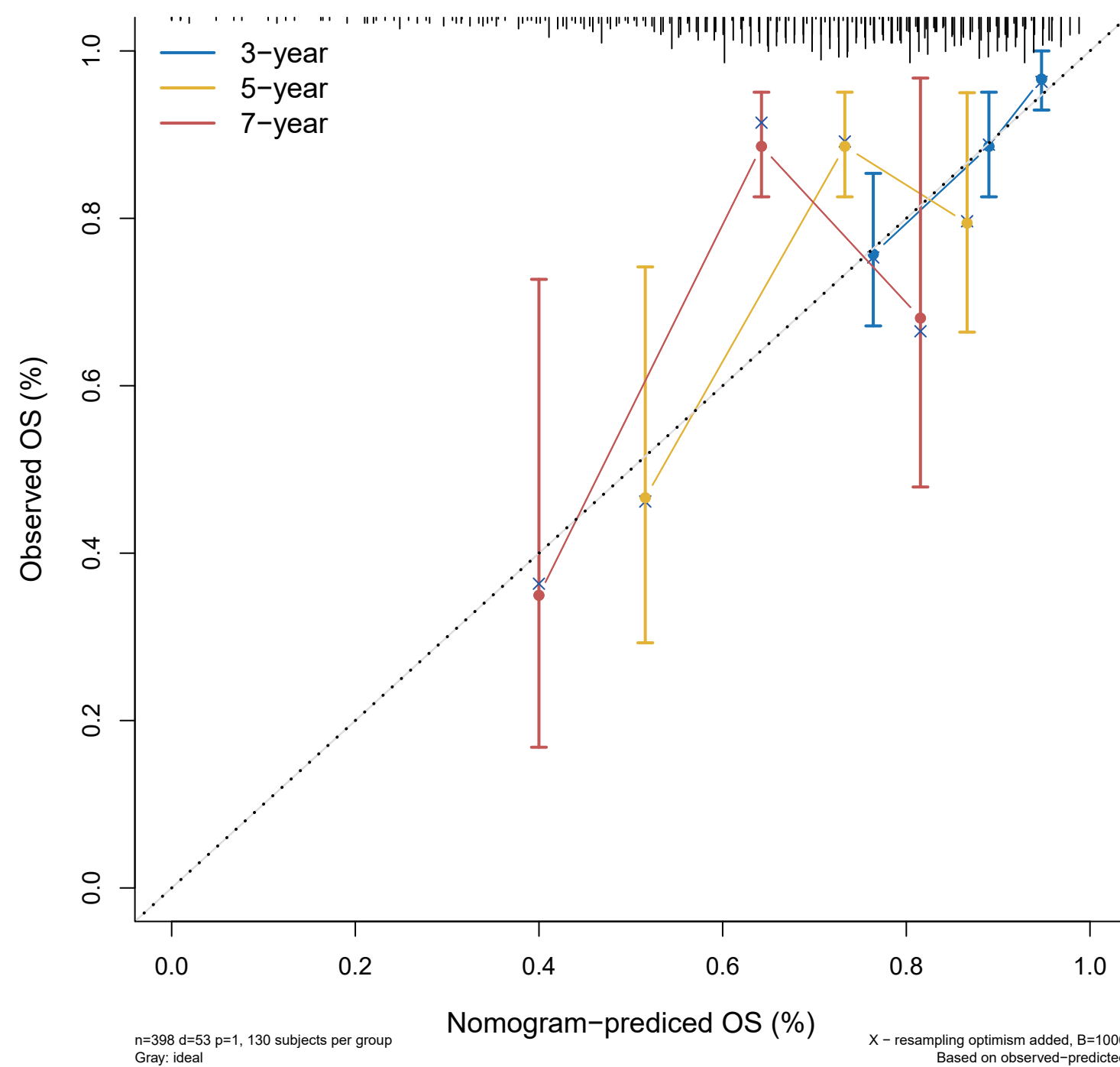**D**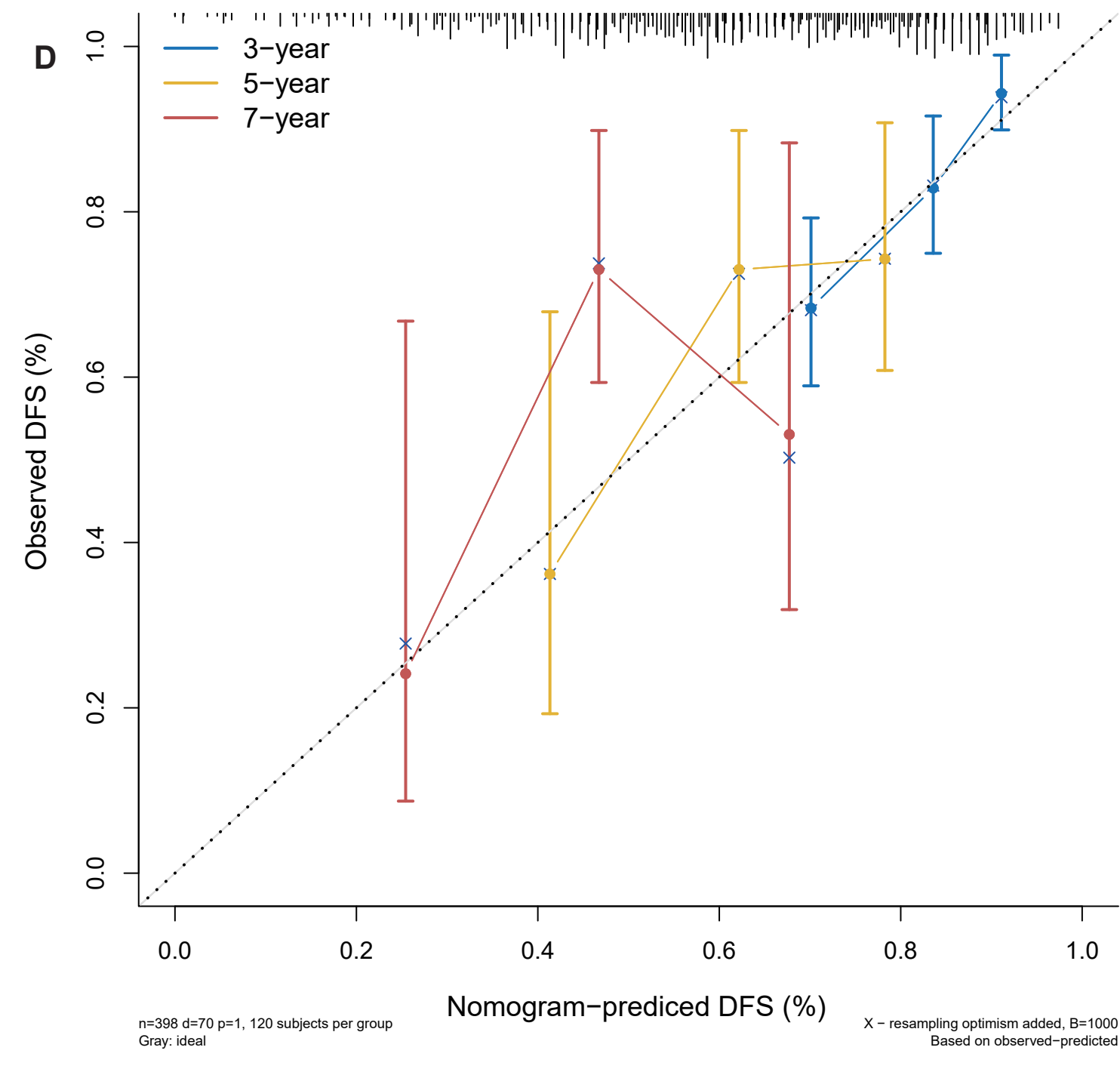

Supplement: Supplementary file 4 [file Image4.pdf]

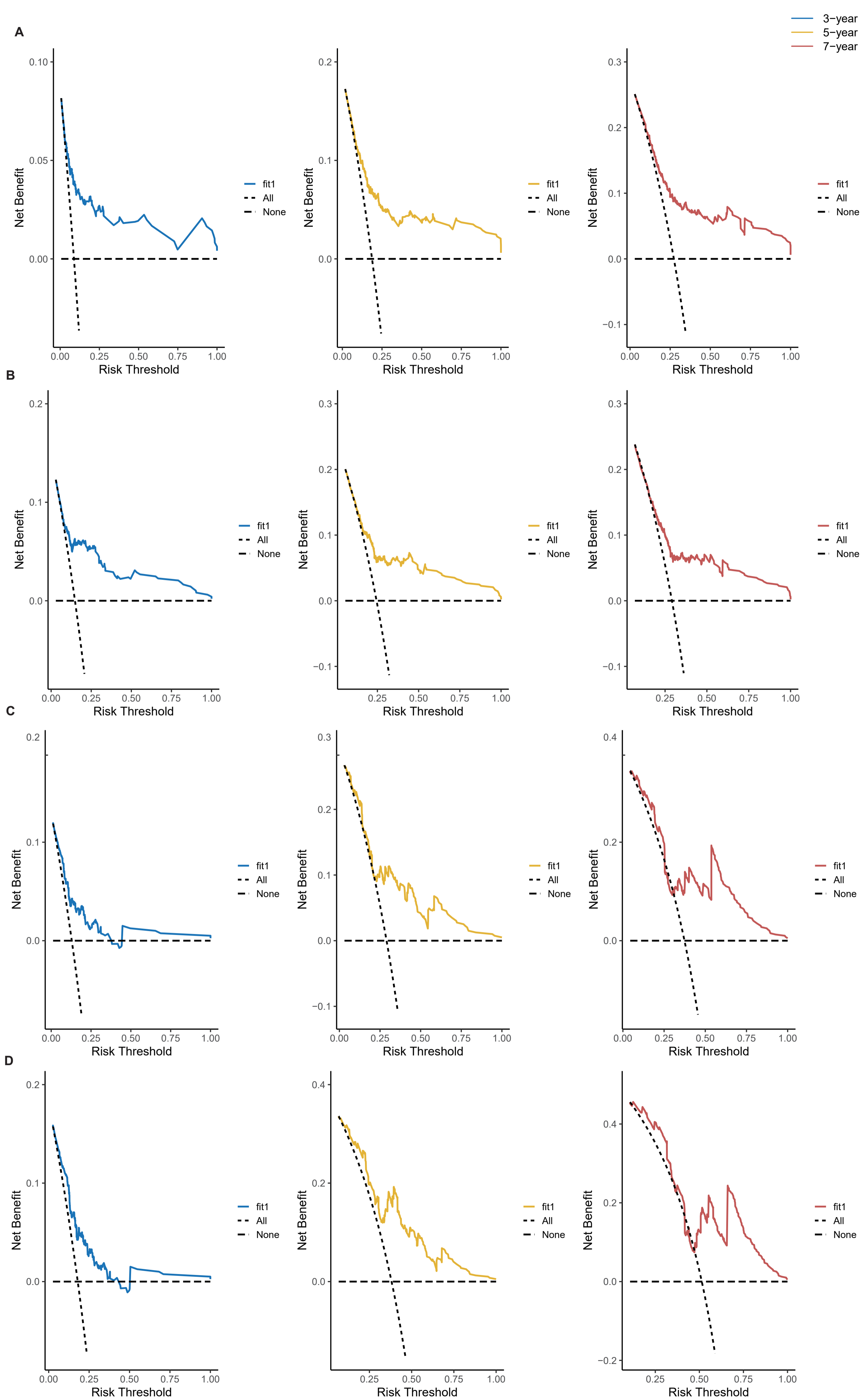

Supplement: Supplementary file 5 [file Image5.pdf]

**A**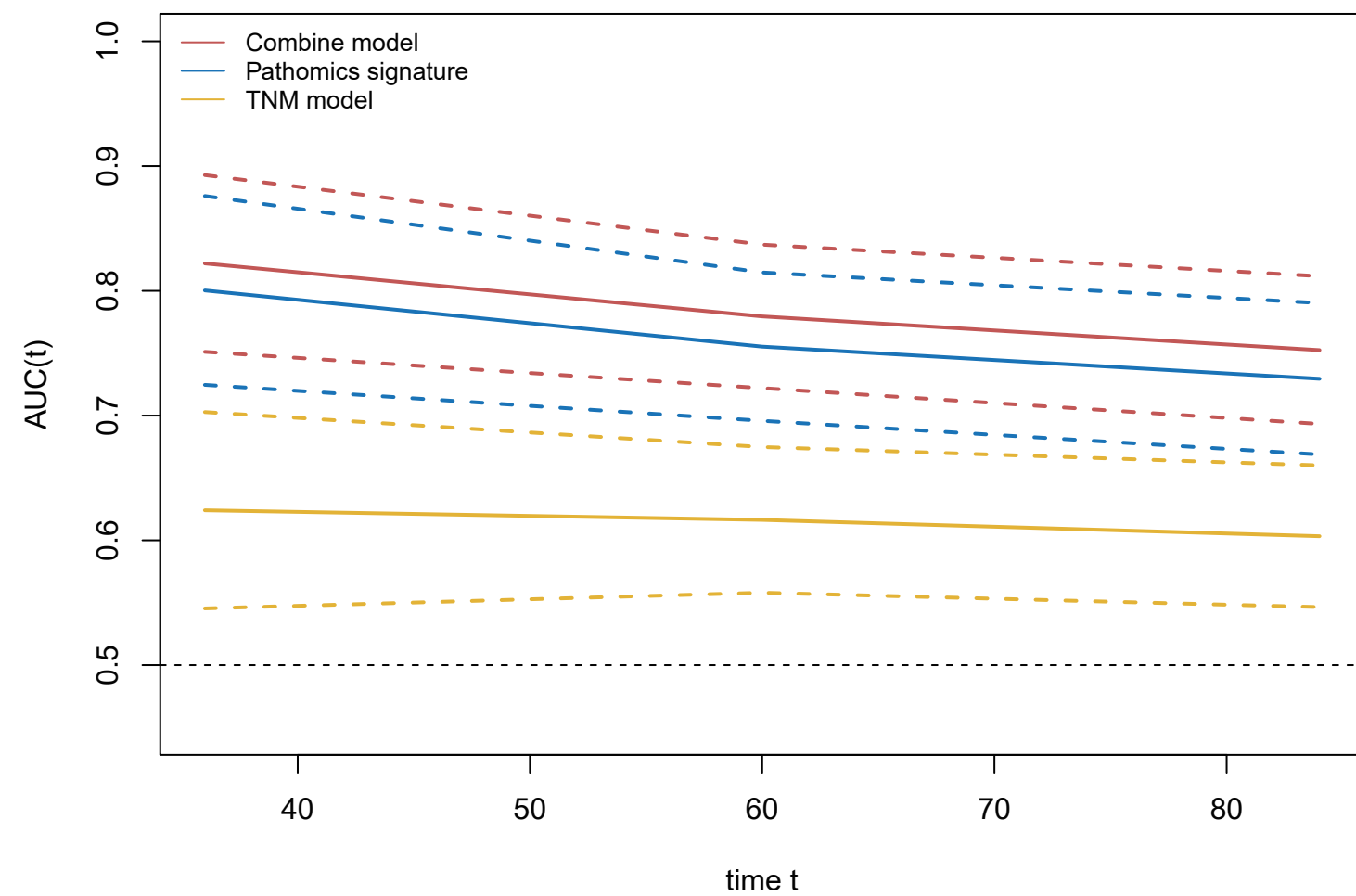**B**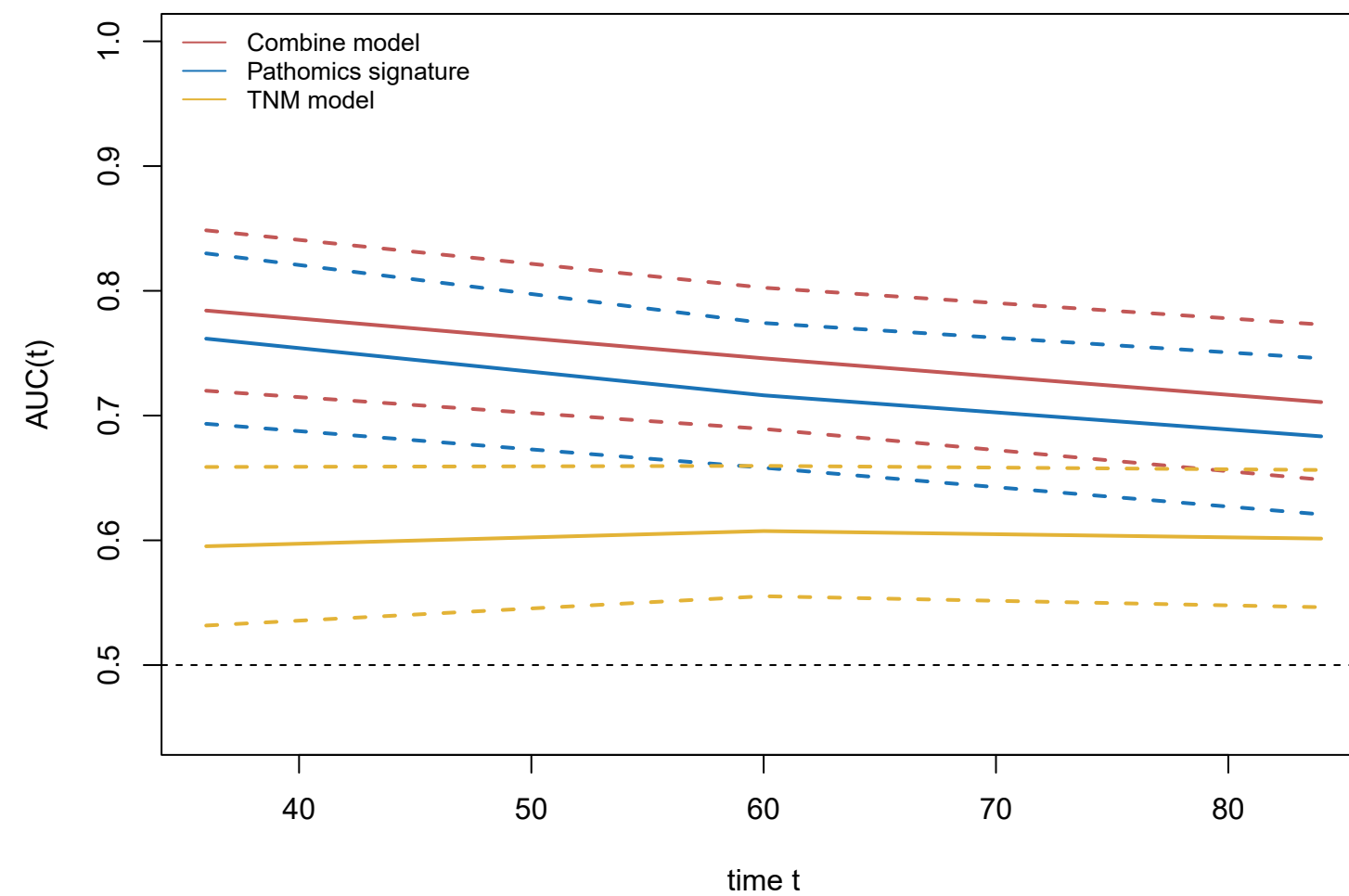**C**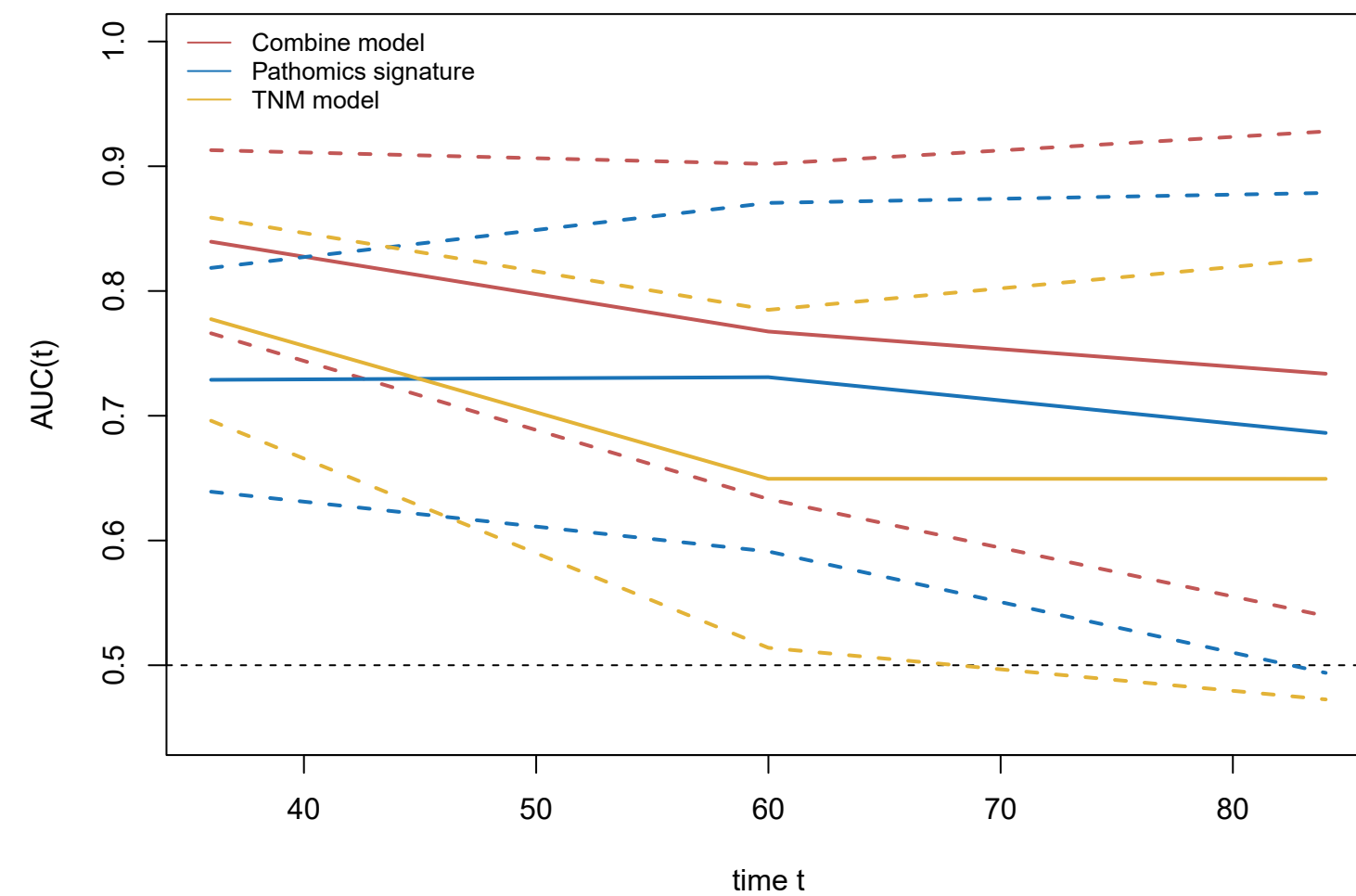**D**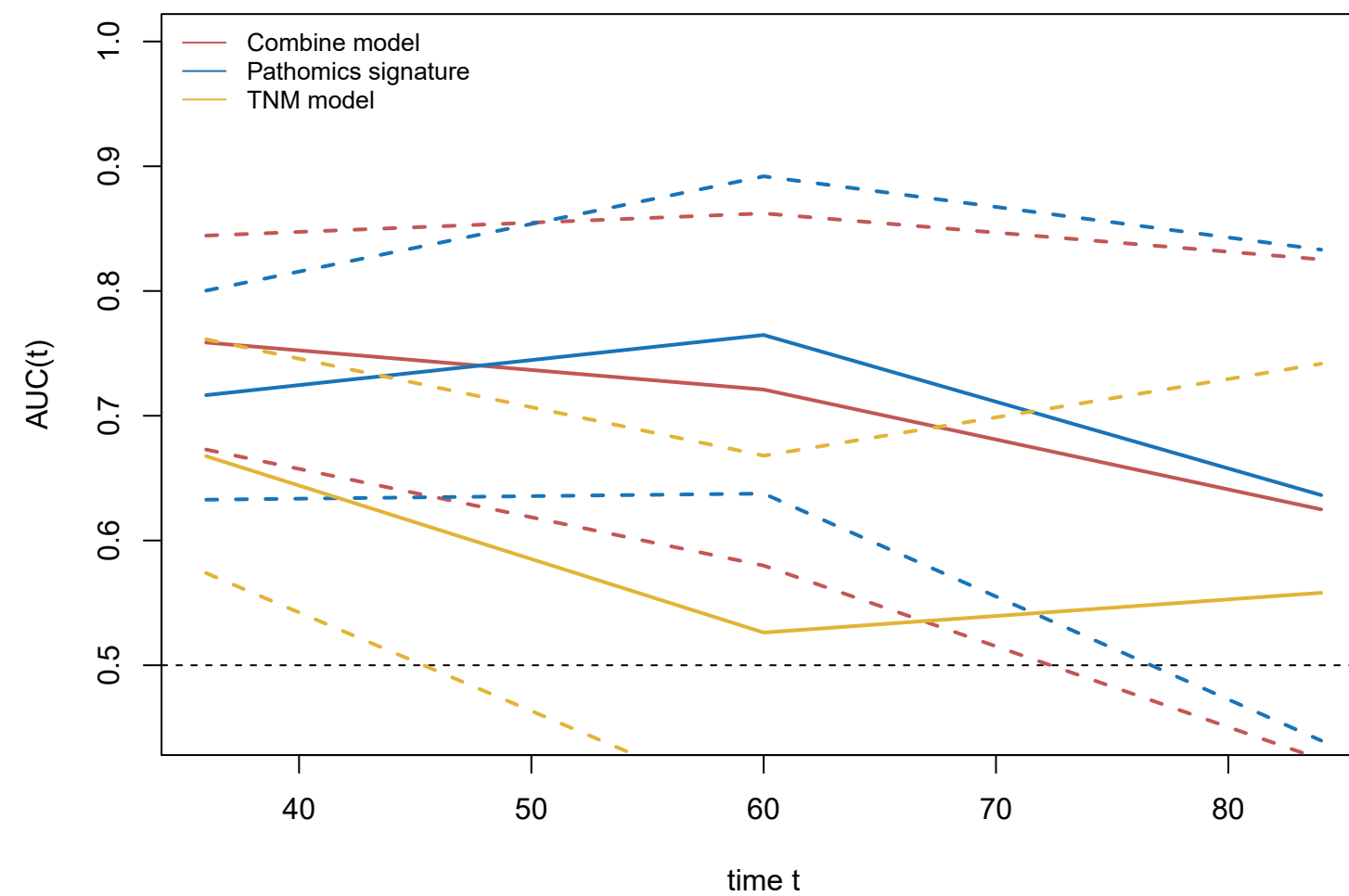

Supplement: Supplementary file 6 [file Image6.pdf]

**A**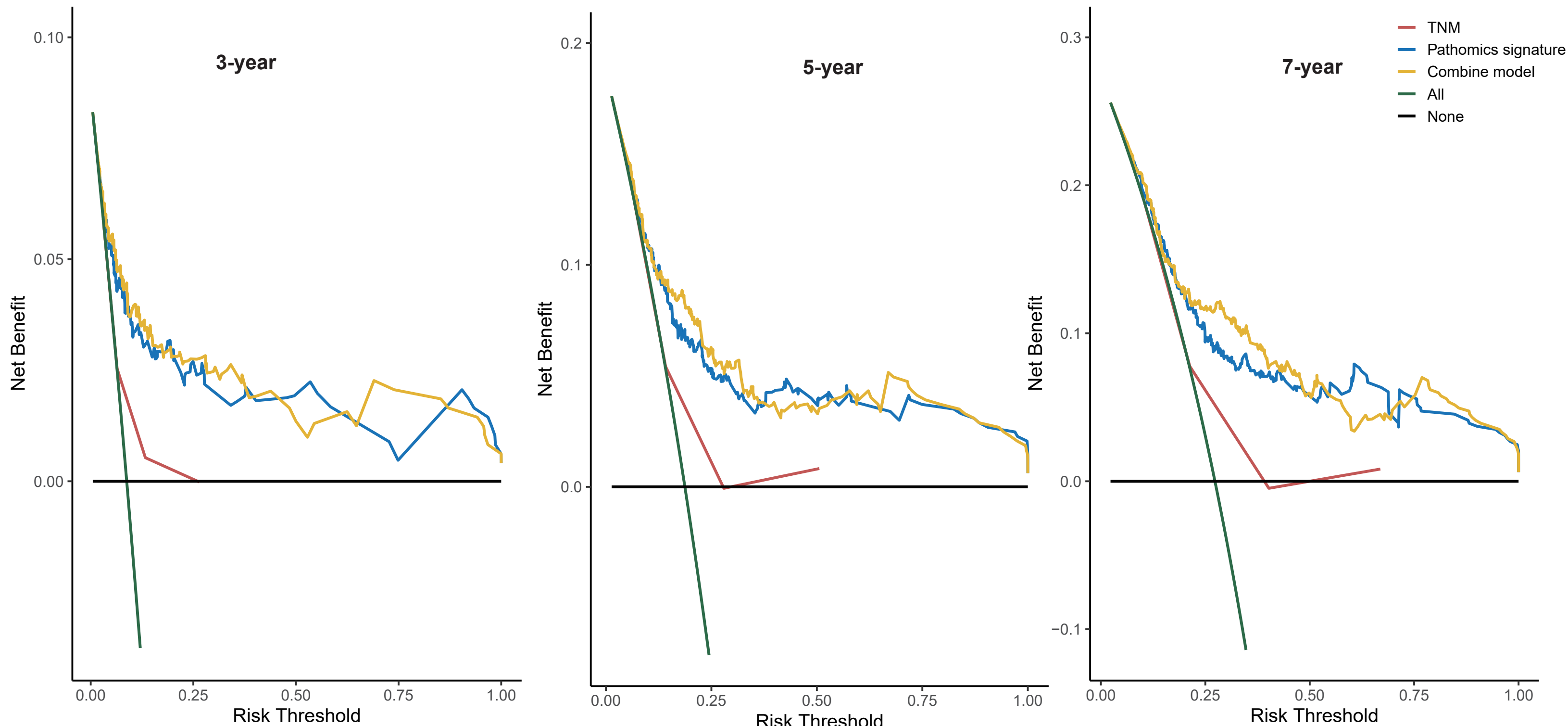**B**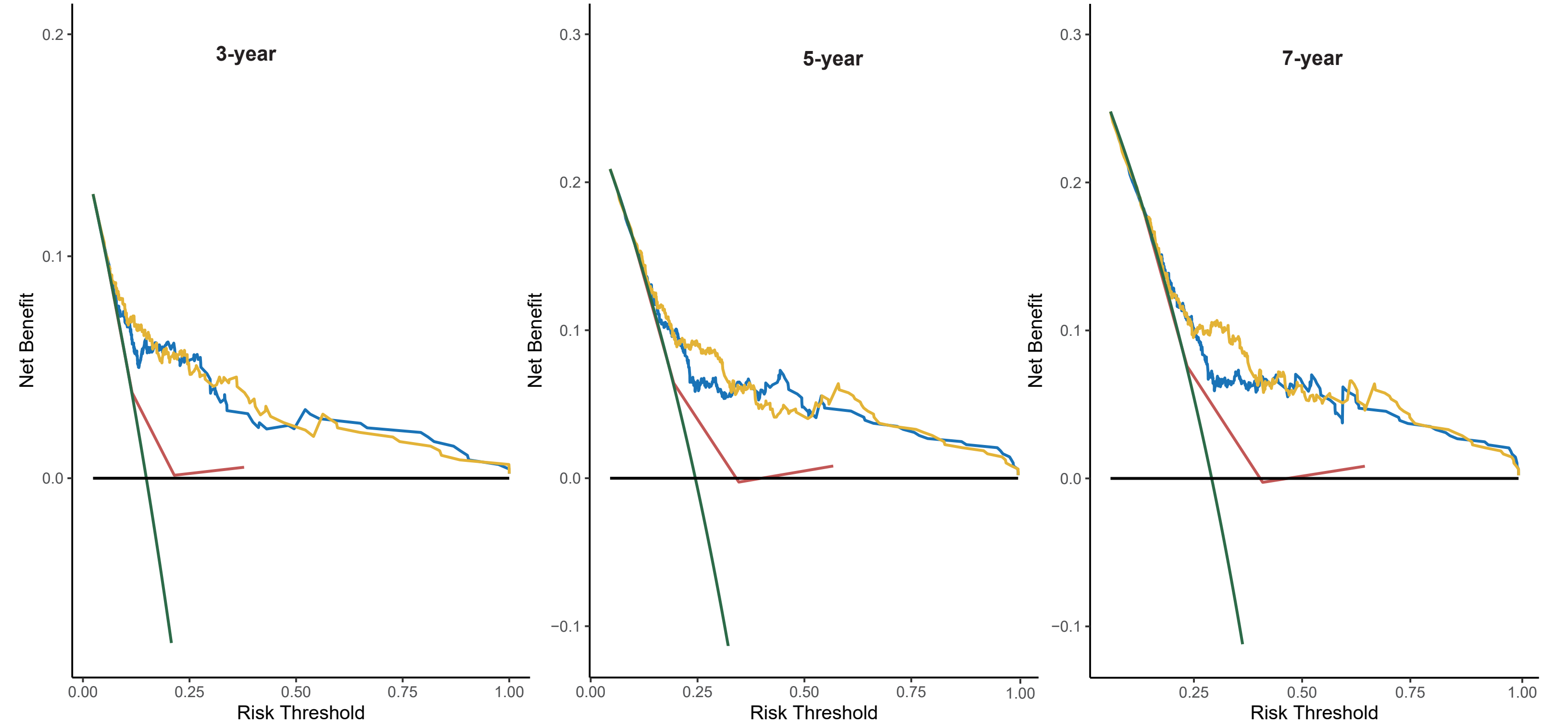**C**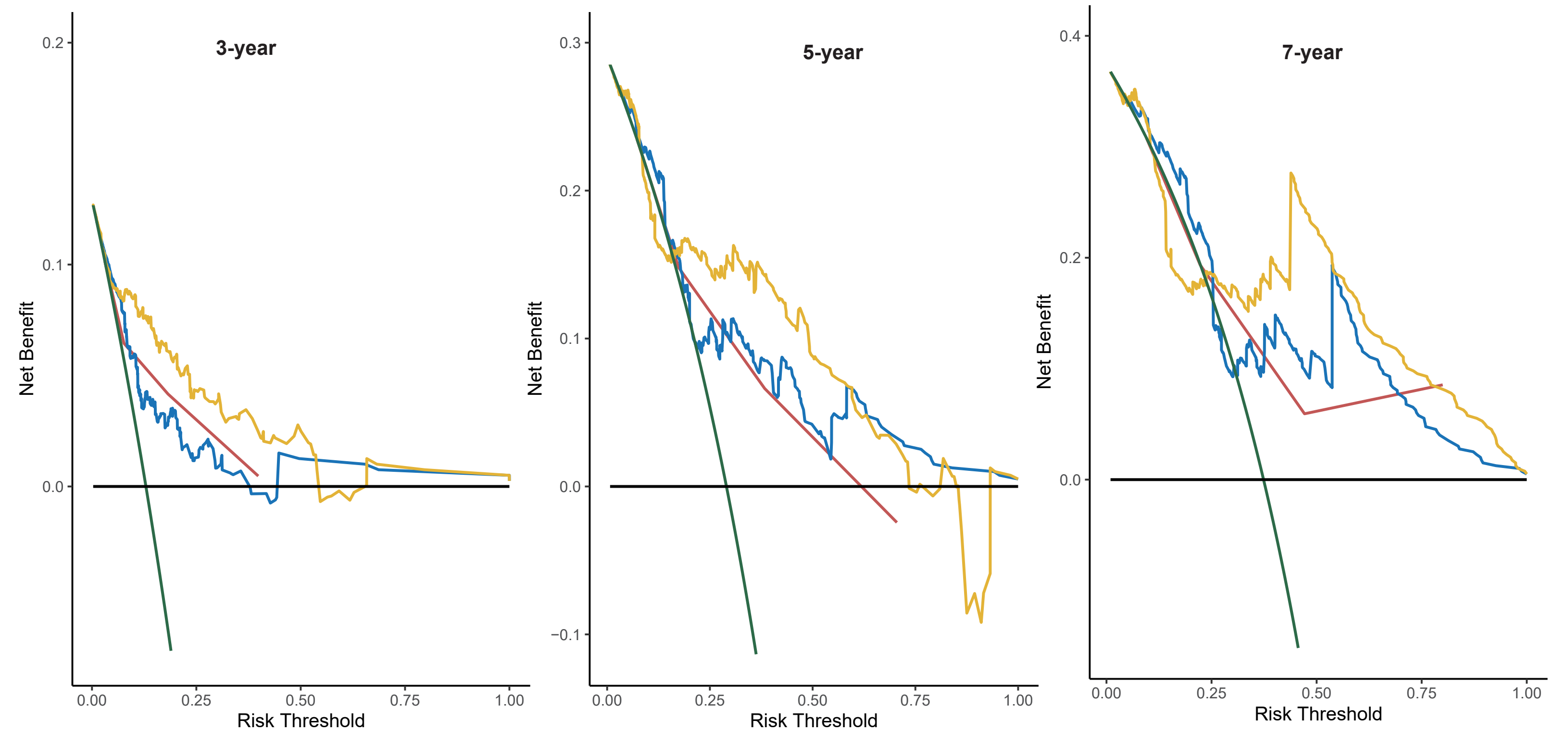**D**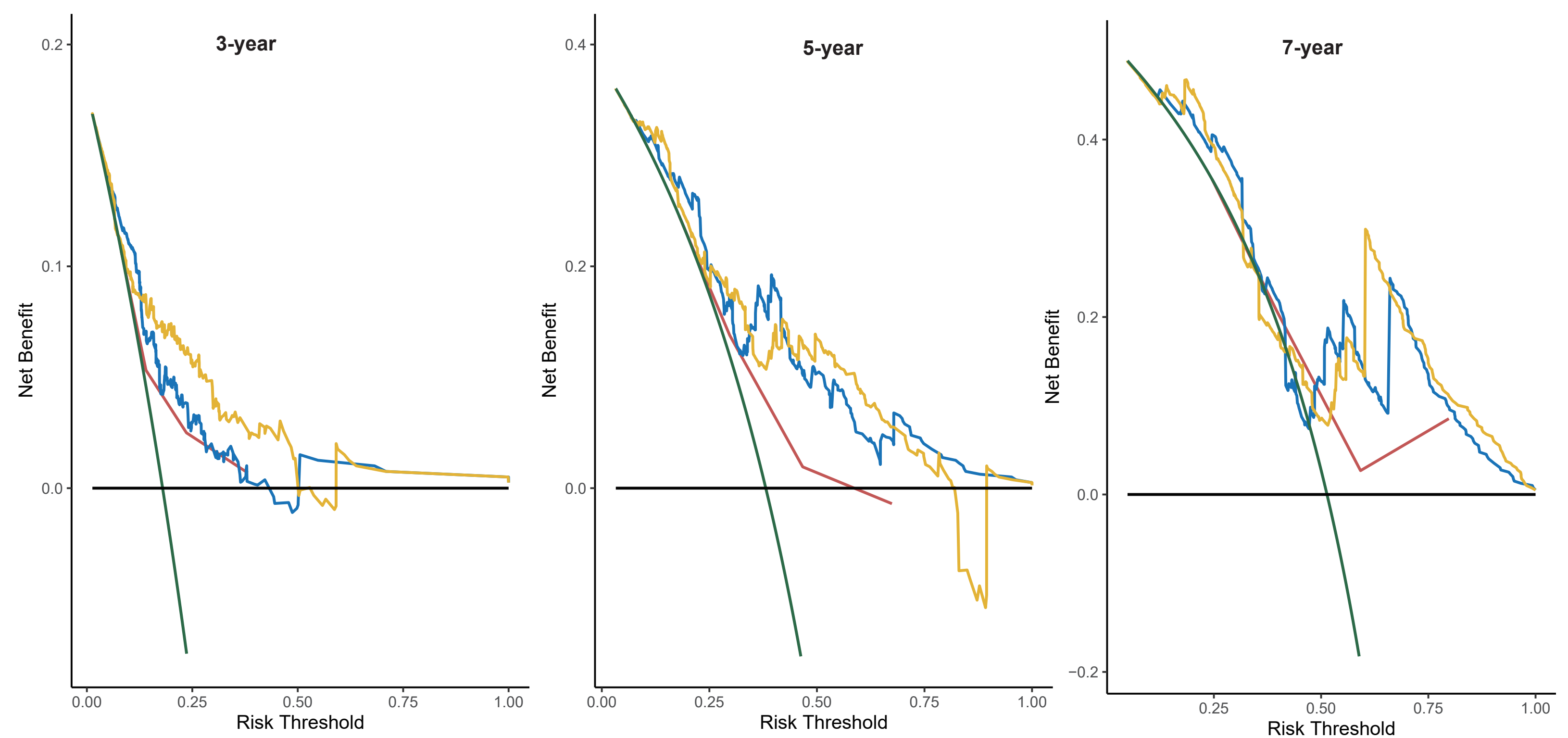

Supplement: Supplementary file 7 [file Image7.pdf]

A

## Chemotherapy

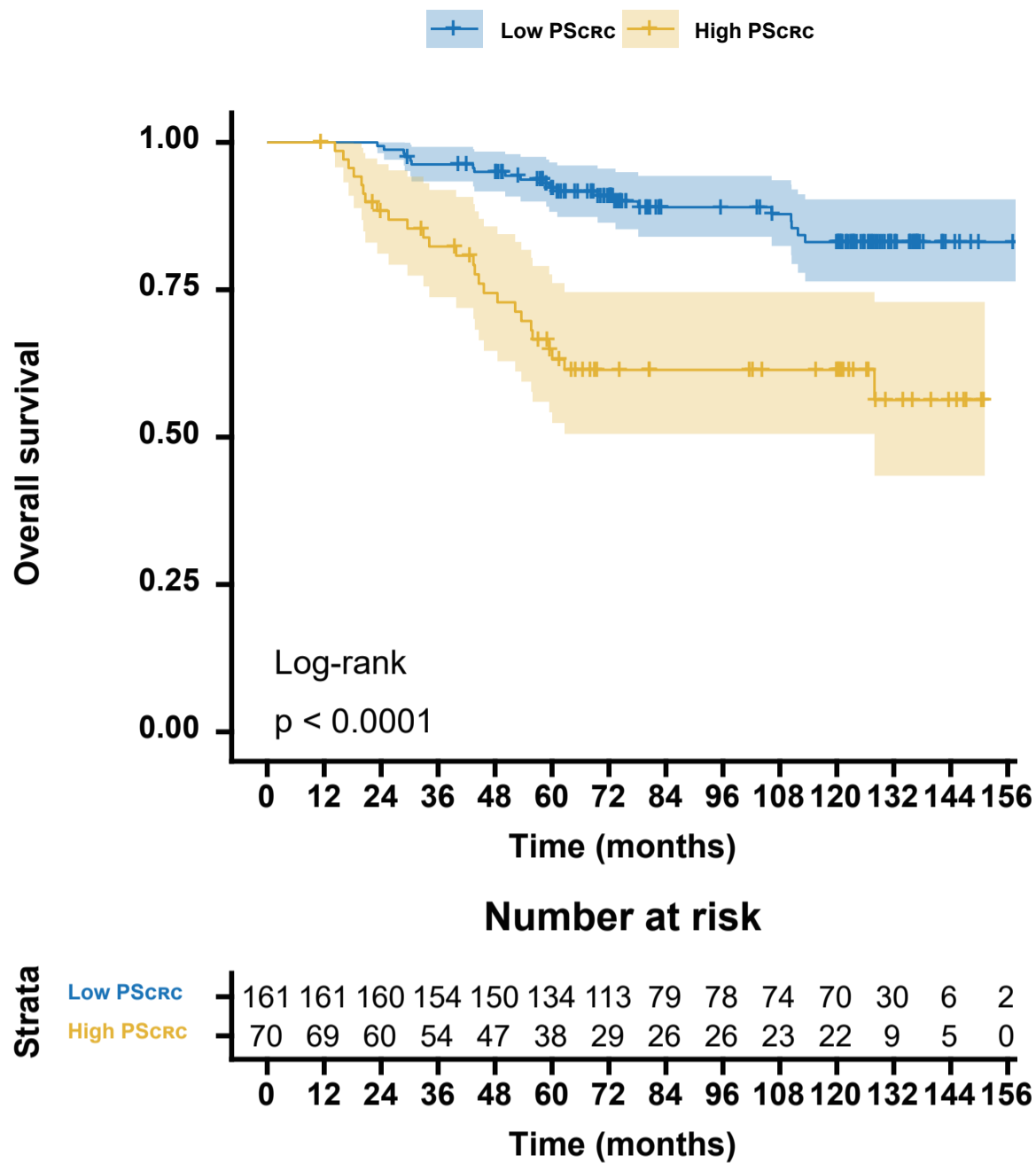

B

## Chemotherapy

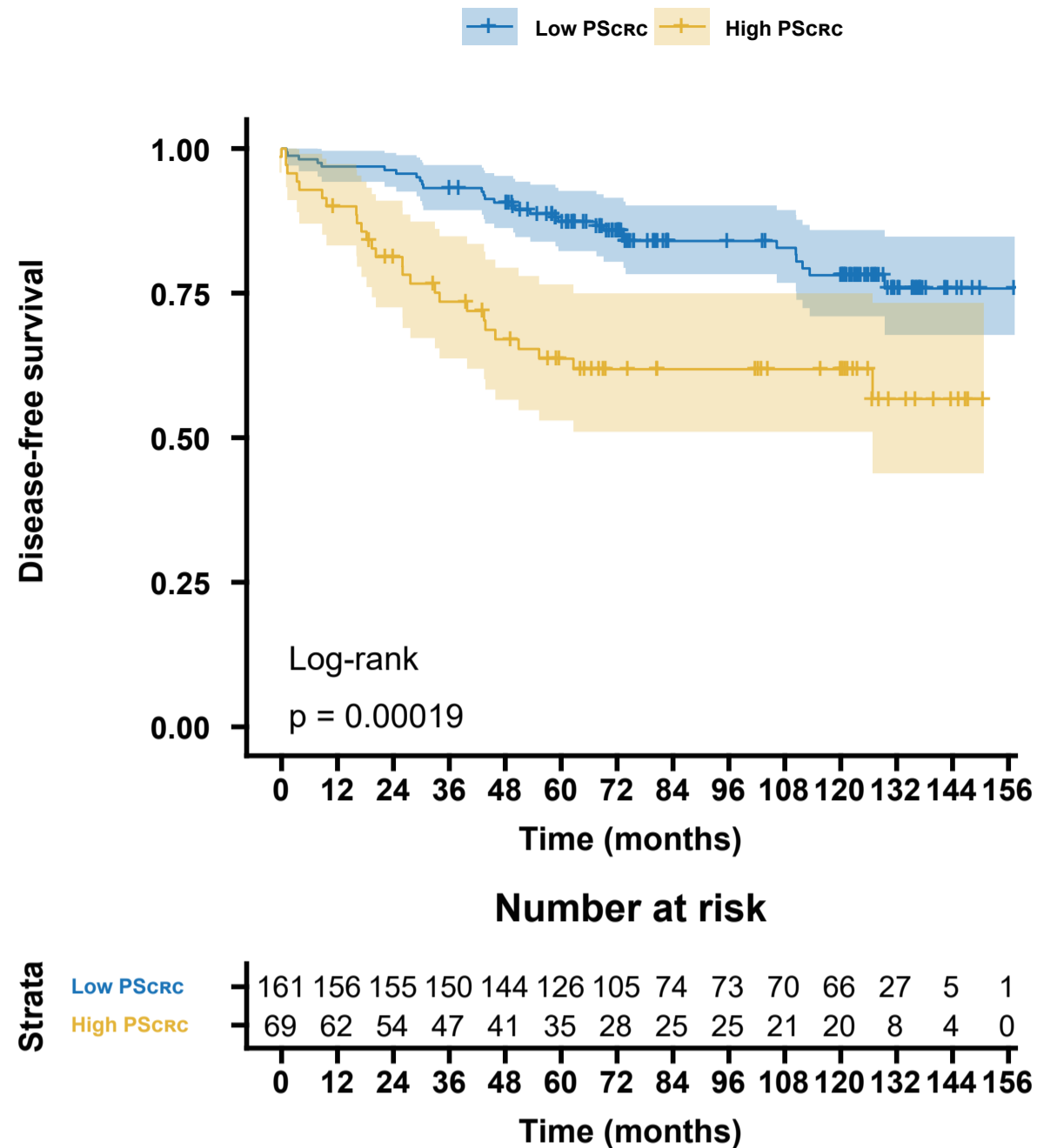

C

## No chemotherapy

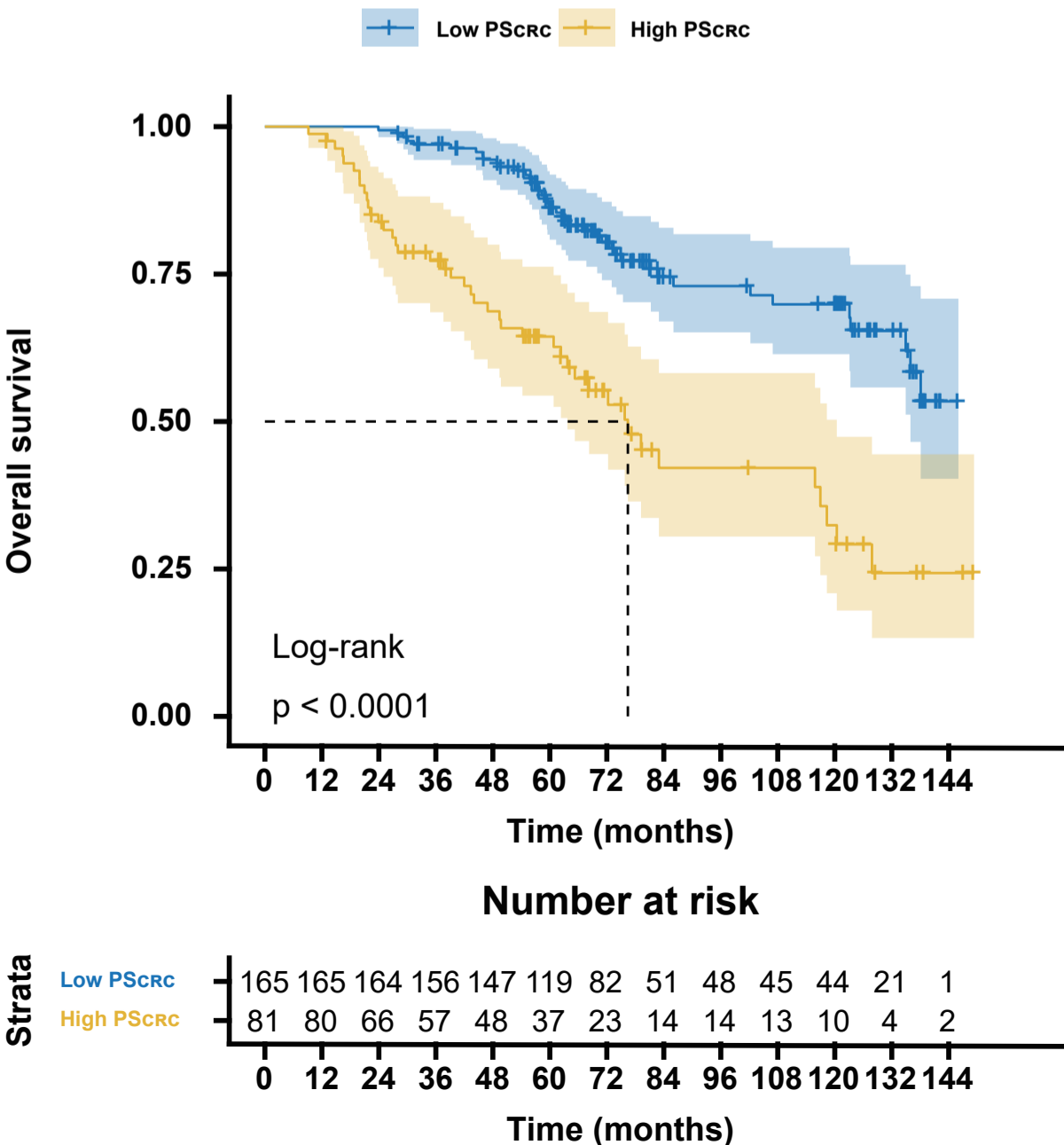

D

## No chemotherapy

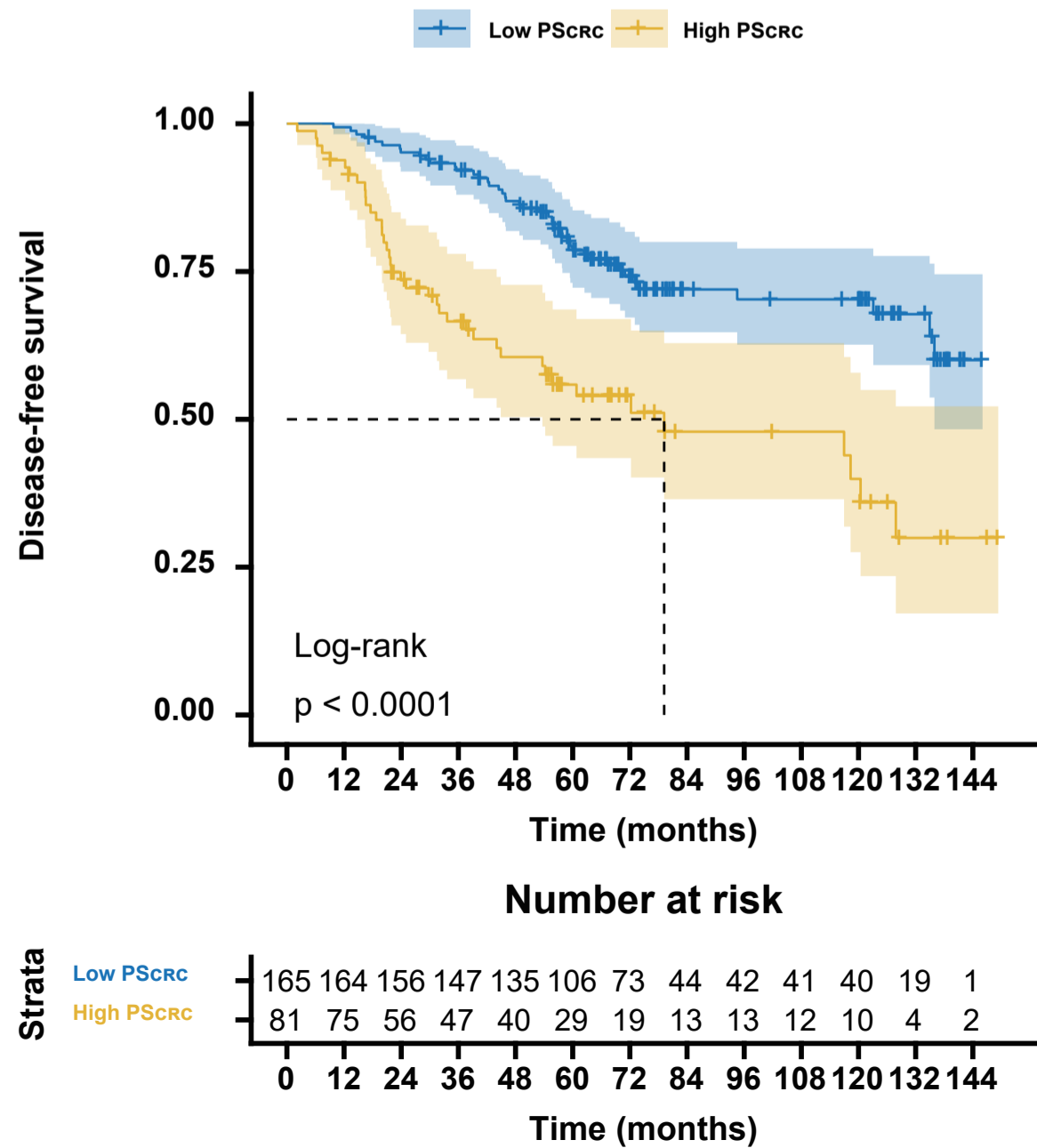

Supplement: Supplementary file 10 [file Image10.pdf]

A

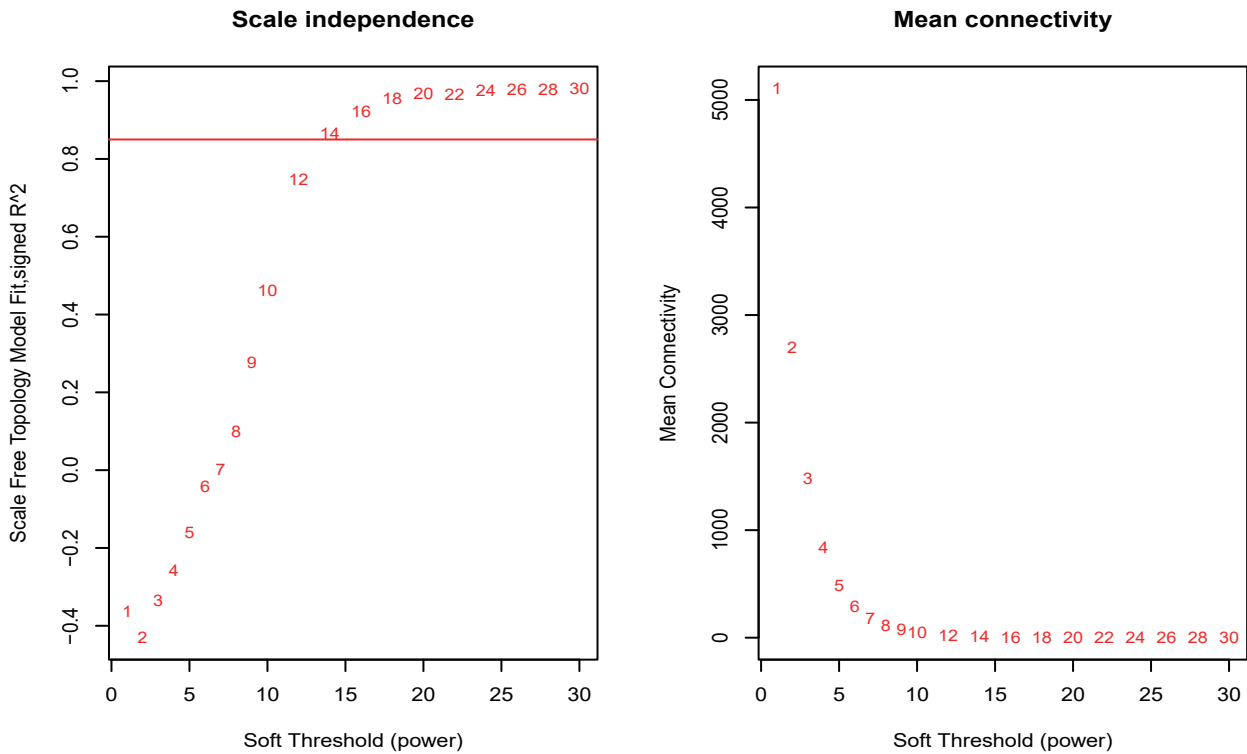

B

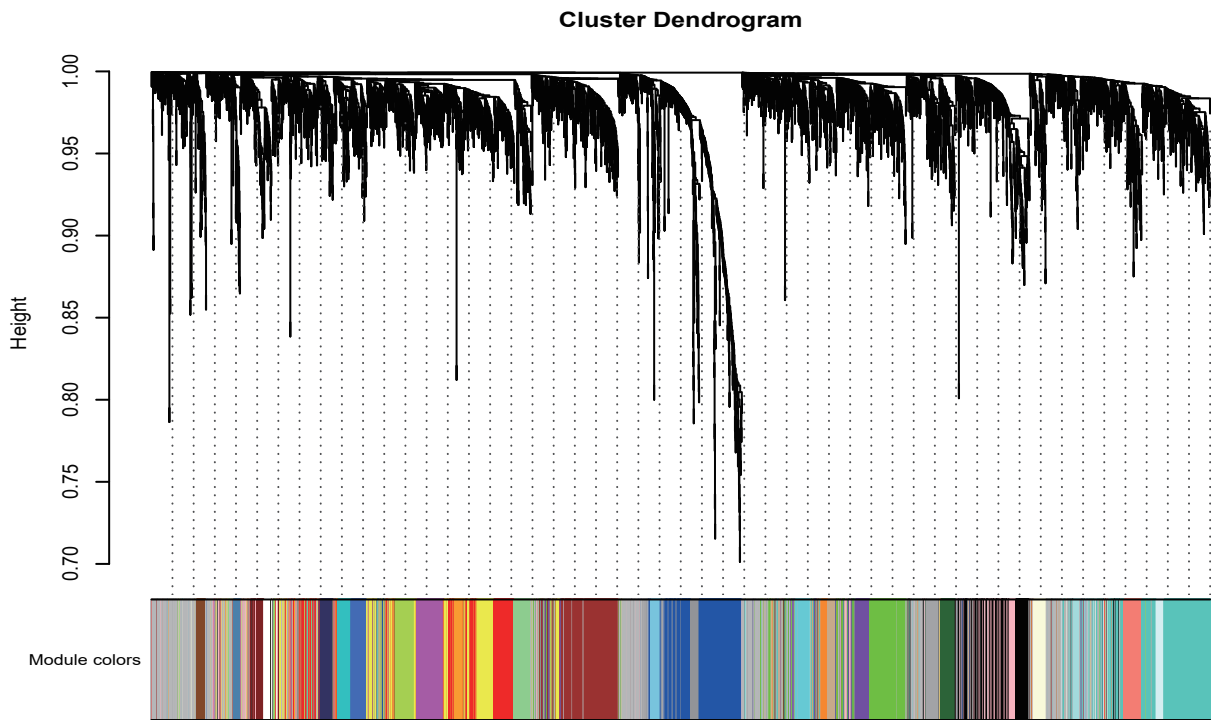

C

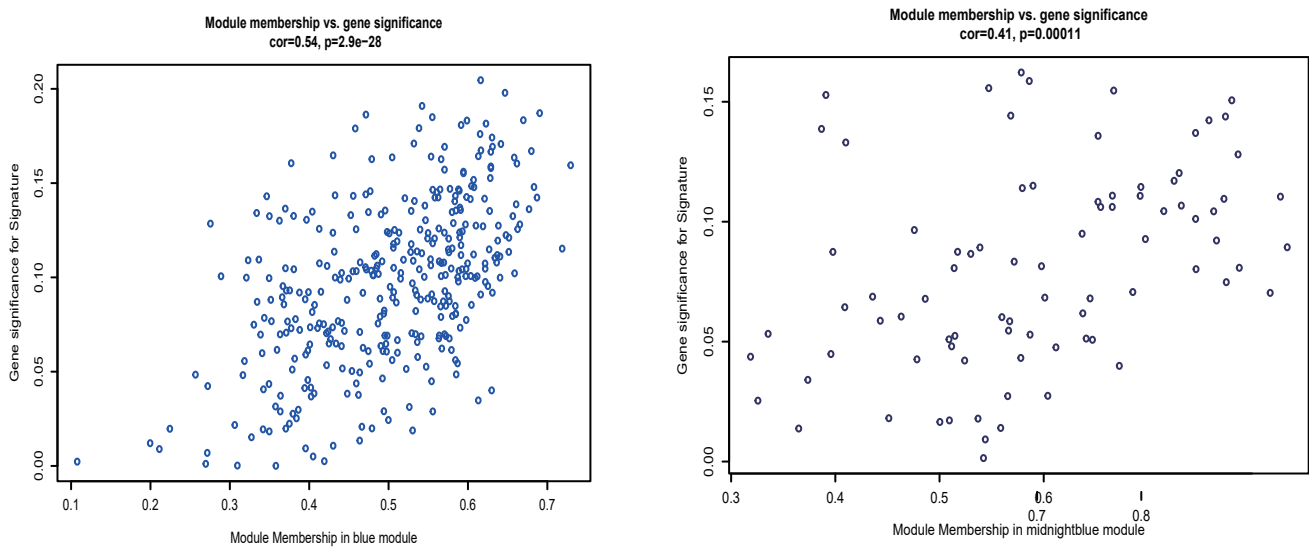

Supplement: Supplementary file 11 [file Image11.pdf]
