# Supplementary material for: Development and validation of a deep learning-based pathomics signature for prognosis and chemotherapy benefits in colorectal cancer: a retrospective multicenter cohort study
Source: Front Immunol. 2025 Jul 8;16:1602909. doi: 10.3389/fimmu.2025.1602909 (PMC12280904; doi:10.3389/fimmu.2025.1602909)
Supplement: Supplementary file 8 [file Image8.pdf]

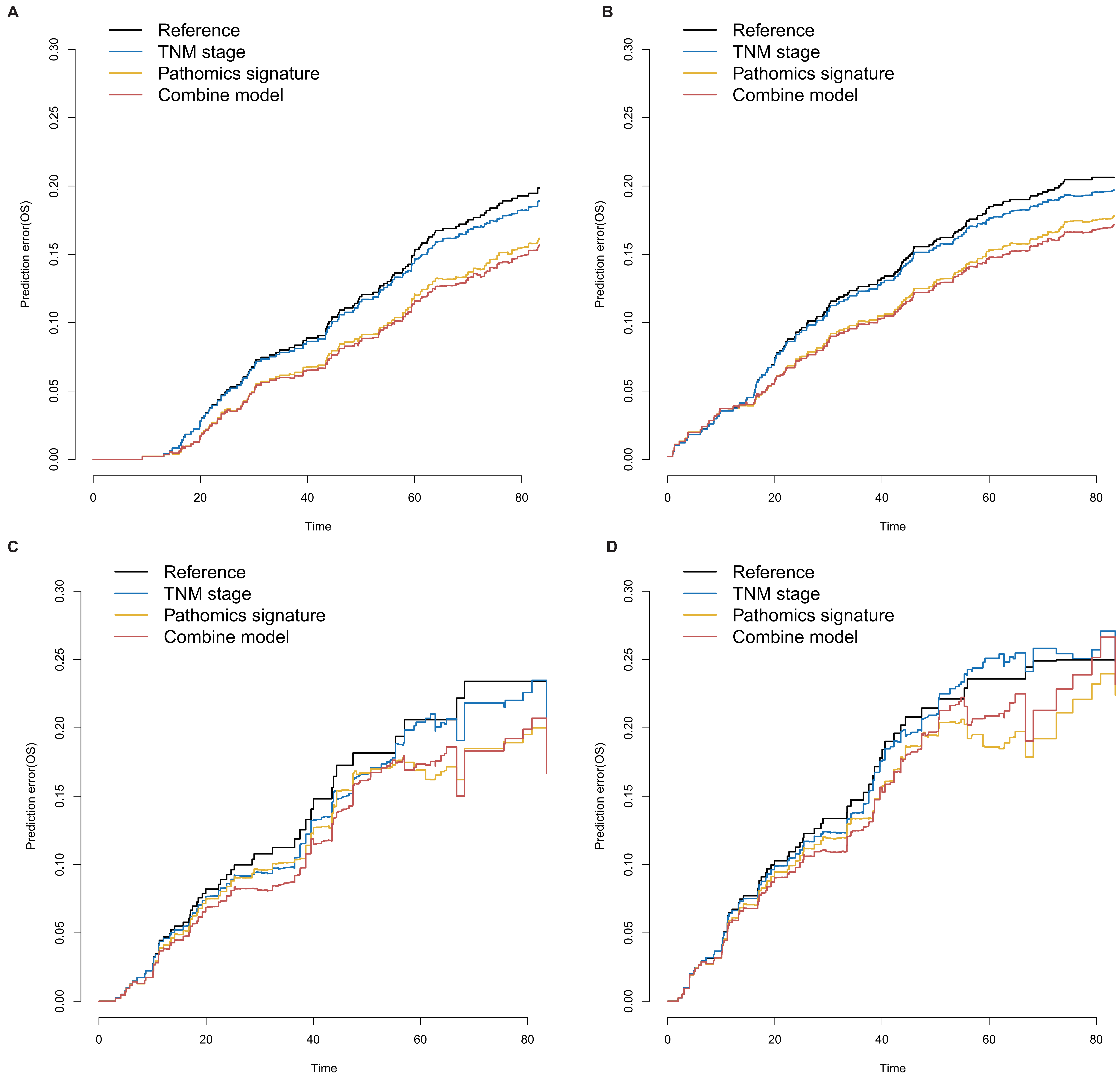
A
B
C
D
Reference
TNM stage
Pathomics signature
Combine model
Prediction error(OS)
Time
0.00
0.05
0.10
0.15
0.20
0.25
0.30
0
20
40
60
80
