# Supplementary material for: Development and validation of a deep learning-based pathomics signature for prognosis and chemotherapy benefits in colorectal cancer: a retrospective multicenter cohort study
Source: Front Immunol. 2025 Jul 8;16:1602909. doi: 10.3389/fimmu.2025.1602909 (PMC12280904; doi:10.3389/fimmu.2025.1602909)
Supplement: Supplementary file 9 [file Image9.pdf]

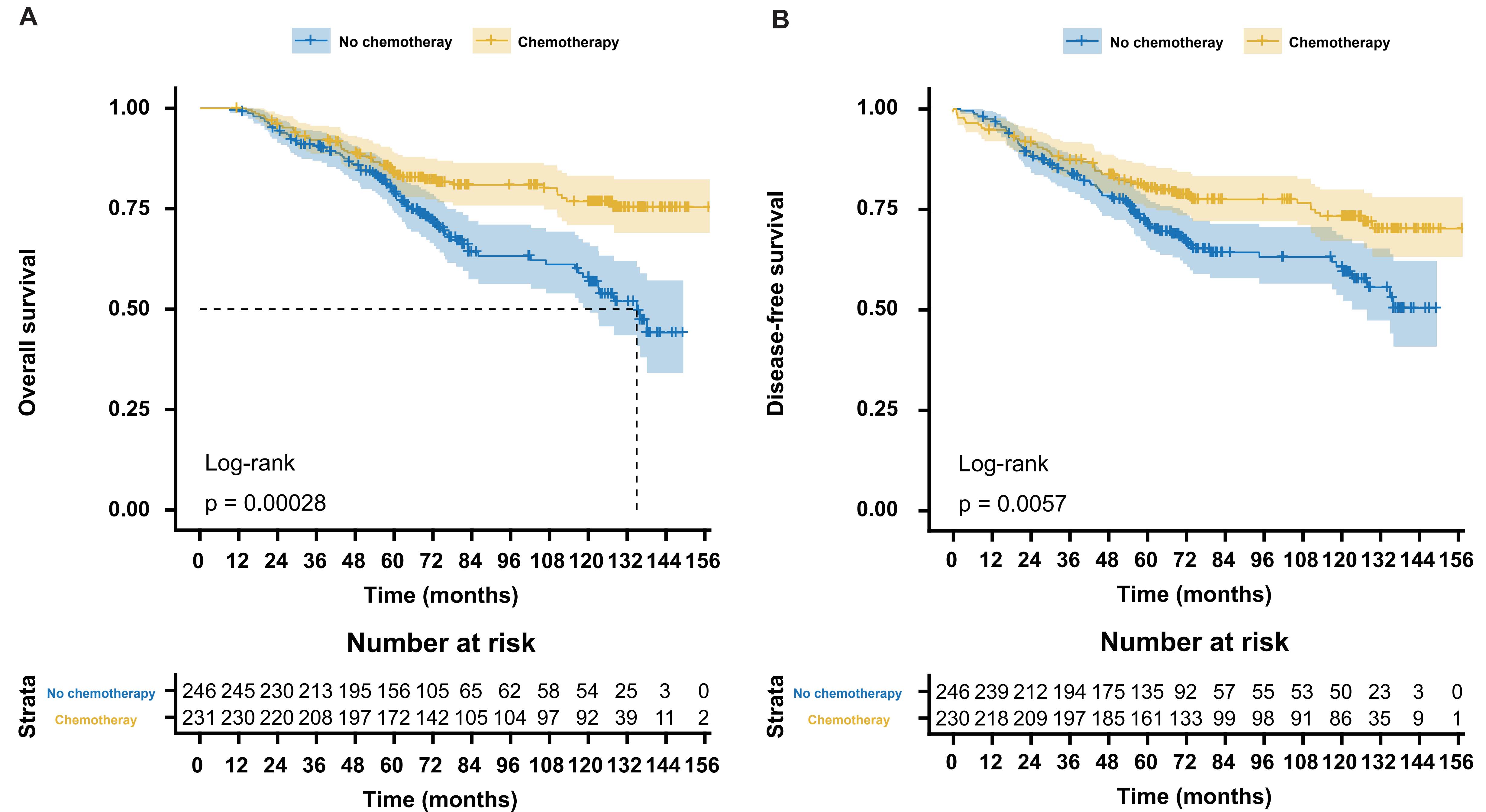
A
No chemotherapy
Chemotherapy
Overall survival
1.00
0.75
0.50
0.25
0.00
Log-rank
p = 0.00028
0 12 24 36 48 60 72 84 96 108 120 132 144 156
Time (months)
Number at risk
Strata
No chemotherapy 246 245 230 213 195 156 105 65 62 58 54 25 3 0
Chemotheray 231 230 220 208 197 172 142 105 104 97 92 39 11 2
0 12 24 36 48 60 72 84 96 108 120 132 144 156
Time (months)
B
No chemotherapy
Chemotherapy
Disease-free survival
1.00
0.75
0.50
0.25
0.00
Log-rank
p = 0.0057
0 12 24 36 48 60 72 84 96 108 120 132 144 156
Time (months)
Number at risk
Strata
No chemotherapy 246 239 212 194 175 135 92 57 55 53 50 23 3 0
Chemotheray 230 218 209 197 185 161 133 99 98 91 86 35 9 1
0 12 24 36 48 60 72 84 96 108 120 132 144 156
Time (months)
