# Supplementary material for: Development and validation of a deep learning-based pathomics signature for prognosis and chemotherapy benefits in colorectal cancer: a retrospective multicenter cohort study
Source: Front Immunol. 2025 Jul 8;16:1602909. doi: 10.3389/fimmu.2025.1602909 (PMC12280904; doi:10.3389/fimmu.2025.1602909)
Supplement: Supplementary file 12 [file DataSheet1.docx]

**Figure legend of images**

**Image 1.** Pathomics feature selection using a LASSO-Cox regression model. **(A)**Tuning parameter (λ) selection for the LASSO-Cox regression model via 10-fold cross-validation. **(B)** Profiles of coefficients from the LASSO-Cox regression

model of the extracted pathomics features. LASSO, least absolute shrinkage and selection operator.

**Image 2.** The distribution of the pathomics signature with the corresponding survival status in the training and validation cohorts. **(A)** The distribution of the PS_CRC_, DFS status and the profiles of the selected pathomics features in the training cohort. **(B)** The distribution of the PS_CRC_, DFS status and the profiles of the selected pathomics features in the validation cohort. DFS disease-free survival, PS_CRC_, pathomics signature of colorectal cancer.

**Image 3.** Time-dependent ROC curves of the pathomics signature. **(A, B)** 3-, 5- and 7-year time-dependent ROC curves of the pathomics signature for OS and DFS prediction in the training cohort. **(C, D)** 3-, 5- and 7-year time-dependent ROC curves of the pathomics signature for OS and DFS prediction in the validation cohort. OS, overall survival; DFS, disease-free survival.

**Image 4.** The calibration curves of the pathomics signature. **(A, B)** The calibration curves of the pathomics signature for 3-, 5- and 7-year OS and DFS in the training cohort. **(C, D)** The calibration curves of the pathomics signature for 3-, 5- and 7-year OS and DFS in the validation cohort. OS, overall survival; DFS, disease-free survival.

**Image 5.** Decision curve analysis of the pathomics signature. **(A, B)** 3-, 5- and 7-year decision curve analysis of OS and DFS in the training cohort. **(C, D)** 3-, 5- and 7-year decision curve analysis of OS and DFS in the validation cohort. OS, overall survival; DFS, disease-free survival.

**Image 6.** Time-dependent ROC curves of different models for OS and DFS. **(A, B)** Time-dependent ROC curves of different models for OS and DFS in the training cohort. **(C, D)** Time-dependent ROC curves of different models for OS and DFS in the validation cohort. OS, overall survival; DFS, disease-free survival; TNM, tumor-node-metastasis.

**Image 7.** Decision curve analysis of different models for OS and DFS. **(A, B)** 3-, 5- and 7-year decision curve analysis of different models for predicting OS and DFS in the training cohort. **(C, D)** 3-, 5- and 7-year decision curve analysis of different models for predicting OS and DFS in the validation cohort. OS, overall survival; DFS, disease-free survival; TNM, tumor-node-metastasis.

**Image 8.** Prediction error curves of different models for OS and DFS. **(A, B)** Prediction error curves of different models for predicting OS and DFS in the training cohort in the training cohort. **(C, D)** Prediction error curves of different models for predicting OS and DFS in the validation cohort. In the prediction error curves, lower prediction errors indicate higher model accuracy. DFS, disease-free survival; OS, overall survival; TNM, tumor-node-metastasis.

**Image 9.** Kaplan–Meier analyses of OS and DFS according to adjuvant chemotherapy. **(A)** The OS rate difference between the CT and non-CT patients. **(B)** The DFS rate difference between the CT and non-CT patients. CT, chemotherapy; DFS, disease-free survival; OS, overall survival.

**Image 10.** Kaplan-Meier analysis of OS and DFS in patients with or without

adjuvant chemotherapy according to the pathomics signature. **(A, B)** The OS rate and DFS rate difference between the high- and low- PS_CRC_ patients with adjuvant chemotherapy. **(C, D)** The OS rate and DFS rate difference between the high- and low- PS_CRC_ patients without adjuvant chemotherapy. OS overall survival, DFS disease-free survival, PS_CRC_, pathomics signature of colorectal cancer.

**Image 11.** WGCNA for the pathomics signature-related gene modules. **(A)** Identification of the soft threshold according to the standard of the scale-free network. The red line represents the threshold line of 0.85. **(B)** Hierarchical dendrogram of the co-expression modules identified by WGCNA. **(C)** Intra-modular analysis for the pathomics signature-related modules. The scatterplot shows gene significance vs. module membership in the blue and midnightblue modules. WGCNA, weighted correlation network analysis.
