# Supplementary material for: Development and validation of a deep learning-based pathomics signature for prognosis and chemotherapy benefits in colorectal cancer: a retrospective multicenter cohort study
Source: Front Immunol. 2025 Jul 8;16:1602909. doi: 10.3389/fimmu.2025.1602909 (PMC12280904; doi:10.3389/fimmu.2025.1602909)
Supplement: Supplementary file 13 [file SupplementaryFile1.docx]

**Table S1.** Characteristics of the patients in the training and validation cohorts

| **Variables** | **Patients, No. (%)** | | ***P*** value |
| --- | --- | --- | --- |
|  | **Training cohort (n = 485)** | **Validation cohort (n = 398)** |  |
| **Age, years** |  |  | **< 0.001** |
| Mean (SD) | 59.38 (11.57) | 65.53 (12.56) |  |
| ≤ 65 | 340 (70.10%) | 181 (45.48%) |  |
| > 65 | 145 (29.90%) | 217 (54.52%) |  |
| **Sex** |  |  | 0.151 |
| Male | 274 (56.49%) | 211 (53.02%) |  |
| Female | 211 (43.51%) | 187 (46.98%) |  |
| **Tumor location** |  |  | 0.087 |
| Left side | 213 (43.92%) | 104 (26.13%) |  |
| Right side | 212 (43.71%) | 138 (34.67%) |  |
| Rectum | 60 (12.37%) | 156 (39.20%) |  |
| **VELI** |  |  | **< 0.001** |
| No | 440 (90.72%) | 247 (62.06%) |  |
| Yes | 45 (9.28%) | 151 (37.94%) |  |
| **Perineural invasion** |  |  | **0.004** |
| No | 398 (82.06%) | 352 (88.44%) |  |
| Yes | 87 (17.94%) | 46 (11.56%) |  |
| **Lymph node harvest** |  |  | **< 0.001** |
| ≤ 12 | 106 (21.86%) | 43 (10.80%) |  |
| > 12 | 379 (78.14%) | 355 (89.20%) |  |
| **Depth of invasion** |  |  | **< 0.001** |
| T1 | 1 (0.21%) | 13 (3.27%) |  |
| T2 | 15 (3.09%) | 78 (19.60%) |  |
| T3 | 265 (54.64%) | 269 (67.59%) |  |
| T4 | 204 (42.06%) | 38 (9.55%) |  |
| **Lymph node metastasis** |  |  | **< 0.001** |
| N0 | 329 (67.84%) | 242 (60.80%) |  |
| N1 | 117 (24.12%) | 97 (24.37%) |  |
| N2 | 39 (8.04%) | 59 (14.82%) |  |
| **Distant metastasis** |  |  | **< 0.001** |
| M0 | 481 (99.18%) | 364 (91.46%) |  |
| M1 | 4 (0.82%) | 34 (8.54%) |  |
| **TNM stage** |  |  | **< 0.001** |
| Stage I | 4 (0.82%) | 80 (20.10%) |  |
| Stage II | 324 (66.80%) | 159 (39.95%) |  |
| Stage III | 153 (31.55%) | 125 (31.41%) |  |
| Stage IV | 4 (0.82%) | 34 (8.54%) |  |
| **MSI status** |  |  | 0.063 |
| MSI-High | 17 (3.51%) | 56 (14.07%) |  |
| MSS | 60 (12.37%) | 334 (83.92%) |  |
| NA | 408 (84.12%) | 8 (2.01%) |  |
| **Survial status** |  |  | < 0.001 |
| Alive | 352 (72.58%) | 345 (86.68%) |  |
| Death | 133 (27.42%) | 53 (13.32%) |  |

TNM, tumor-node-metastasis; VELI, venous emboli and/or lymphatic invasion; MSI, microsatellite instable; MSS, microsatellite stable; NA, not available

**Table S2.** Characteristics of the patients according to the pathomics signature

in the training and validation cohorts

| **Variables** | **Training cohort (n = 485)** | | ***P*** value | **Validation cohort (n = 398)** | | ***P*** value |
| --- | --- | --- | --- | --- | --- | --- |
|  | **High (n = 155)** | **Low (n = 330)** |  | **High (n = 171)** | **Low (n = 227)** |  |
| **Age, years** |  |  |  |  |  |  |
| Mean (SD) | 59.22 (11.73) | 59.72 (11.25) | 0.663 | 65.08 (12.71) | 65.86 (12.47) | 0.54 |
| **Sex, No. (%)** |  |  |  |  |  |  |
| Male | 91 (58.71%) | 183 (55.45%) | 0.5 | 93 (54.39%) | 118 (51.98%) | 0.634 |
| Female | 64 (41.29%) | 147 (44.55%) |  | 78 (45.61%) | 109 (48.02%) |  |
| **Tumor location, No. (%)** |  |  |  |  |  |  |
| Left side | 16 (10.32%) | 44 (13.33%) | 0.572 | 48 (28.07%) | 56 (24.67%) | 0.4 |
| Right side | 72 (46.45%) | 141 (42.73%) |  | 53 (30.99%) | 85 (37.44%) |  |
| Rectum | 67 (43.23%) | 145 (43.94%) |  | 70 (40.94%) | 86 (37.89%) |  |
| **VELI, No. (%)** |  |  |  |  |  |  |
| No | 137 (88.39%) | 303 (91.82%) | 0.225 | 103 (60.23%) | 144 (63.44%) | 0.515 |
| Yes | 18 (11.61%) | 27 (8.18%) |  | 68 (39.77%) | 83 (36.56%) |  |
| **Perineural invasion, No. (%)** |  |  |  |  |  |  |
| No | 120 (77.42%) | 278 (84.24%) | 0.068 | 146 (85.38%) | 206 (90.75%) | 0.097 |
| Yes | 35 (22.58%) | 52 (15.76%) |  | 25 (14.62%) | 21 (9.25%) |  |
| **Lymph node harvest, No. (%)** |  |  |  |  |  |  |
| ≤ 12 | 31 (20.00%) | 75 (22.73%) | 0.498 | 25 (14.62%) | 18 (7.93%) | **0.033** |
| > 12 | 124 (80.00%) | 255 (77.27%) |  | 146 (85.38%) | 209 (92.07%) |  |
| **Depth of invasion, No. (%)** |  |  |  |  |  |  |
| T1 | 1 (0.65%) | 0 (0.00%) | 0.303 | 7 (4.09%) | 6 (2.64%) | 0.344 |
| T2 | 4 (2.58%) | 11 (3.33%) |  | 27 (15.79%) | 51 (22.47%) |  |
| T3 | 79 (50.97%) | 186 (56.36%) |  | 119 (69.59%) | 150 (66.08%) |  |
| T4 | 71 (45.81%) | 133 (40.30%) |  | 18 (10.53%) | 20 (8.81%) |  |
| **Lymph node metastasis, No. (%)** |  |  |  |  |  |  |
| N0 | 95 (61.29%) | 234 (70.91%) | **0.03** | 96 (56.14%) | 146 (64.32%) | 0.249 |
| N1 | 41 (26.45%) | 76 (23.03%) |  | 46 (26.90%) | 51 (22.47%) |  |
| N2 | 19 (12.26%) | 20 (6.06%) |  | 29 (16.96%) | 30 (13.22%) |  |
| **Distant metastasis, No. (%)** |  |  |  |  |  |  |
| M0 | 152 (98.06%) | 329 (99.70%) | 0.064 | 151 (88.30%) | 213 (93.83%) | 0.051 |
| M1 | 3 (1.94%) | 1 (0.30%) |  | 20 (11.70%) | 14 (6.17%) |  |
| **TNM stage, No. (%)** |  |  |  |  |  |  |
| Stage I | 1 (0.65%) | 3 (0.91%) | 0.056 | 26 (15.20%) | 54 (23.79%) | 0.06 |
| Stage II | 93 (60.00%) | 231 (70.00%) |  | 68 (39.77%) | 91 (40.09%) |  |
| Stage III | 58 (37.42%) | 95 (28.79%) |  | 57 (33.33%) | 68 (29.96%) |  |
| Stage IV | 3 (1.94%) | 1 (0.30%) |  | 20 (11.70%) | 14 (6.17%) |  |
| **Adjuvant chemotherapy, No. (%)** |  |  |  |  |  |  |
| No | 83 (53.55%) | 168 (50.91%) | 0.588 | 4 (28.57%) | 8 (61.54%) | 0.085 |
| Yes | 72 (46.45%) | 162 (49.09%) |  | 10 (71.43%) | 5 (38.46%) |  |
| **MSI status, No. (%)** |  |  |  |  |  |  |
| MSI-H | 14 (73.68%) | 46 (79.31%) | 0.608 | 145 (86.31%) | 189 (85.14%) | 0.743 |
| MSS | 5 (26.32%) | 12 (20.69%) |  | 23 (13.69%) | 33 (14.86%) |  |

TNM, tumor-node-metastasis; VELI, venous emboli and/or lymphatic invasion; MSI, microsatellite instable; MSS, microsatellite stable

**Table S3.** Univariate and multivariate Cox regression analyses of the pathomics signature and clinicopathological characteristics for overall survival and disease-free survival in the validation cohort

| **Variables** | **Samples** | **Overall survival** | | | | **Disease-free survival** | | | |
| --- | --- | --- | --- | --- | --- | --- | --- | --- | --- |
|  |  | **Univariate analysis** | | **Multivariate analysis** | | **Univariate analysis** | | **Multivariate analysis** | |
|  |  | **HR (95% CI)** | P **value** | **HR (95% CI)** | P **value** | **HR (95% CI)** | P **value** | **HR (95% CI)** | P **value** |
| **Age, years** |  | 1.035 (1.011, 1.060) | **0.0042** | 1.053 (1.027, 1.080) | **0.00007** | 1.026 (1.005, 1.047) | **0.013** | 1.033 (1.011, 1.056) | **0.0037** |
| **Sex** |  |  |  |  |  |  |  |  |  |
| Male | 211 | Reference |  |  |  | Reference |  |  |  |
| Female | 187 | 1.127 (0.657, 1.933) | 0.66 |  |  | 1.145 (0.715, 1.834) | 0.57 | 1.453 (0.810, 2.608) | 0.21 |
| **Tumor location** |  |  |  |  |  |  |  |  |  |
| Left side |  | Reference |  |  |  | Reference |  |  |  |
| Right side |  | 0.747 (0.344, 1.622) | 0.46 |  |  | 0.897 (0.470, 1.710) | 0.74 |  |  |
| Rectum |  | 1.351 (0.683, 2.671) | 0.39 |  |  | 1.143 (0.624, 2.092) | 0.67 |  |  |
| **VELI** |  |  |  |  |  |  |  |  |  |
| No | 247 | Reference |  |  |  | Reference |  |  |  |
| Yes | 151 | 1.923 (1.116, 3.312) | **0.018** | 1.543 (0.759, 3.138) | 0.23 | 1.508 (0.940, 2.419) | 0.089 |  |  |
| **Perineural invasion** |  |  |  |  |  |  |  |  |  |
| No | 352 | Reference |  |  |  | Reference |  |  |  |
| Yes | 46 | 1.468 (0.660, 3.265) | 0.35 |  |  | 1.187 (0.567, 2.484) | 0.65 |  |  |
| **Lymph node harvest** |  |  |  |  |  |  |  |  |  |
| ≤ 12 | 43 | Reference |  |  |  | Reference |  |  |  |
| > 12 | 355 | 0.361 (0.184, 0.707) | **0.003** | 3.819 (1.738, 8.390) | **0.00085** | 0.364 (0.202, 0.658) | **0.0008** | 3.313 (1.735, 6.326) | **0.00028** |
| **Depth of invasion** |  |  |  |  |  |  |  |  |  |
| T1-2 | 91 | Reference |  |  |  | Reference |  |  |  |
| T3 | 269 | 4.974 (1.194, 20.728) | **0.028** | 3.095 (0.718, 13.350) | 0.13 | 2.188 (0.933, 5.134) | 0.072 | 1.835 (0.757, 4.444) | 0.18 |
| T4 | 38 | 21.053 (4.782, 92.687) | **0.00006** | 10.456 (2.202, 49.650) | **0.0031** | 8.342 (3.260, 21.346) | **<0.00001** | 6.139 (2.206, 17.081) | **0.00051** |
| **Lymph node metastasis** |  |  |  |  |  |  |  |  |  |
| N0 | 242 | Reference |  |  |  | Reference |  |  |  |
| N1 | 97 | 2.626 (1.364, 5.057) | **0.0039** | 1.744 (0.817, 3.720) | 0.15 | 1.593 (0.909, 2.792) | 0.103 | 1.003 (0.522, 1.925) | 0.99 |
| N2 | 59 | 4.531 (2.331, 8.809) | **<0.00001** | 2.327 (1.014, 5.338) | **0.046** | 2.582 (1.445, 4.614) | **0.0014** | 1.382 (0.668, 2.862) | 0.38 |
| **Distant metastasis** |  |  |  |  |  |  |  |  |  |
| M0 | 364 | Reference |  |  |  | Reference |  |  |  |
| M1 | 34 | 4.339 (2.347, 8.020) | **<0.00001** | 2.075 (1.036, 4.155) | **0.039** | 2.817 (1.566, 5.066) | **0.00054** | 1.580 (0.808, 3.092) | 0.18 |
| **MSI status** |  |  |  |  |  |  |  |  |  |
| MSI-H | 334 | Reference |  |  |  | Reference |  |  |  |
| MSS | 56 | 1.304 (0.655, 2.600) | 0.45 |  |  | 1.026 (0.538, 1.954) | 0.94 |  |  |
| **Pathomics signature** | 398 | 3.406 (2.480, 4.676) | **<0.00001** | 3.593 (2.480, 5.205) | **<0.00001** | 2.796 (2.129, 3.672) | **<0.00001** | 2.798 (2.055, 3.810) | **<0.00001** |

VELI, venous emboli and/or lymphatic invasion; MSI, microsatellite instable; MSS, microsatellite stable; HR, hazard ratio; CI, confidence interval

**Table S4.** E-Values for the pathomics signature for overall survival and disease-free survival in the training and validation cohorts

| **Pathomics signature** | **Adjusted hazard ratio** | **E-value** | **E-value** |
| --- | --- | --- | --- |
|  | **(95% confidence interva)** | **(Point estimate)** | **(Confidence interval)** |
| **Training cohort (n = 485)** |  |  |  |
| Overall survival | 3.475 (2.785, 4.336) | 4.1 | 3.45 |
| Disease-free survival | 2.235 (1.886, 2.650) | 2.87 | 2.47 |
| **Validation cohort (n = 398)** |  |  |  |
| Overall survival | 3.593 (2.480, 5.205) | 4.21 | 3.14 |
| Disease-free survival | 2.798 (2.055, 3.810) | 3.46 | 2.67 |

**Table S5.** C-indexes for overall survival and disease-free survival in different models

| **Model** | **Training cohort** | | **Validation cohort** | |
| --- | --- | --- | --- | --- |
|  | **C-index (95% CI)** | ***P value*** | **C-index (95% CI)** | ***P value*** |
| **Overall survival** |  |  |  |  |
| TNM stage | 0.590 (0.5460, 0.6341) | **<0.0001** | 0.7189 (0.6469, 0.7908) | **0.033** |
| Pathomics signature | 0.723 (0.6756, 0.7704) | 0.08 | 0.7292 (0.6621, 0.7963) | **<0.0001** |
| Combined model | 0.7404 (0.6942, 0.7866) | Reference | 0.8115 (0.7459, 0.8771) | Reference |
| **Disease-free survival** |  |  |  |  |
| TNM stage | 0.5769 (0.5348, 0.6191) | **<0.0001** | 0.6365 (0.5682, 0.7047) | **0.007** |
| Pathomics signature | 0.6825 (0.6333, 0.7316) | **0.047** | 0.6973 (0.6307, 0.7638) | **0.02** |
| Combined model | 0.7019 (0.6547, 0.7490) | Reference | 0.7329 (0.6638, 0.8020) | Reference |

TNM, tumour -node-metastasis; CI, confidence interval

**Table S6.** Net reclassification improvement and integrated discrimination improvement by adding the pathomics signature to the TNM stage model

| **Model** | **Training cohort** | | | | **Validation cohort** | | | |
| --- | --- | --- | --- | --- | --- | --- | --- | --- |
|  | **Point estimate** | **95% CI** | | ***P value*** | **Point estimate** | **95% CI** | | ***P value*** |
| **Overall survival** |  |  |  |  |  |  |  |  |
| NRI | 0.362 | 0.251 | 0.474 | **<0.0001** | 0.303 | 0.067 | 0.499 | **0.027** |
| IDI | 0.199 | 0.141 | 0.262 | **<0.0001** | 0.109 | 0.037 | 0.2 | **<0.0001** |
| **Disease-free survival** |  |  |  |  |  |  |  |  |
| NRI | 0.288 | 0.169 | 0.417 | **<0.0001** | 0.227 | 0.074 | 0.439 | **0.007** |
| IDI | 0.134 | 0.088 | 0.191 | **<0.0001** | 0.088 | 0.037 | 0.149 | **<0.0001** |

NRI, net reclassification improvement; IDI, integrated discrimination improvement; CI, confidence interval

**Table S7.** Adjuvant chemotherapy interaction with the pathomics signature for survival in stage II and III colorectal cancer patients

| **Pathomics signature** | **Chemotherapy** | | **Overall survival** | | **Disease-free survival** | |
| --- | --- | --- | --- | --- | --- | --- |
|  | **No** | **Yes** | **HR (95% CI)** | ***P* value for interaction** | **HR (95% CI)** | ***P* value for interaction** |
| **Low** | 165 (67.073%) | 81 (32.927%) | 2.961 (2.268, 3.866) | **0.0133** | 2.288 (1.853, 2.825) | **0.017** |
| **High** | 161 (69.697%) | 70 (30.303%) | 4.598 (3.169, 6.670) |  | 2.910 (2.080, 4.073) |  |

HR, hazard ratio; CI, confidence interval

**Table S8.** Association of the pathomics signature and tumor microenvironment

| **Cell type** | **Spearman correlation** | | **Wilcoxon test** | **Overall survival** | | **Disease-free survival** | |
| --- | --- | --- | --- | --- | --- | --- | --- |
|  | **Correlation coefficient** | ***P-value*** | ***P-value*** | **HR (95% CI)** | ***P-***value | **HR (95% CI)** | ***P-***value |
| STROMALSCORE_ESTIMATE | -0.003 | 0.9543 | 0.7368 |  |  |  |  |
| IMMUNESCORE_ESTIMATE | -0.0465 | 0.3688 | 0.5767 |  |  |  |  |
| TUMORPURITY_ESTIMATE | 0.0159 | 0.7583 | 0.782 |  |  |  |  |
| T_CELLS_MCPCOUNTER | -0.0076 | 0.8827 | 0.8205 |  |  |  |  |
| CD8_T_CELLS_MCPCOUNTER | 0.0463 | 0.3702 | 0.1056 |  |  |  |  |
| CYTOTOXIC_LYMPHOCYTES_MCPCOUNTER | -0.0946 | 0.0668 | 0.1697 |  |  |  |  |
| B_LINEAGE_MCPCOUNTER | -0.0473 | 0.3606 | 0.3962 |  |  |  |  |
| NK_CELLS_MCPCOUNTER | -0.0996 | 0.0536 | 0.0242 |  |  |  |  |
| MONOCYTIC_LINEAGE_MCPCOUNTER | 0.0584 | 0.2585 | 0.4092 |  |  |  |  |
| MYELOID_DENDRITIC_CELLS_MCPCOUNTER | 0.0175 | 0.7356 | 0.8911 |  |  |  |  |
| NEUTROPHILS_MCPCOUNTER | -0.0308 | 0.5519 | 0.6693 |  |  |  |  |
| ENDOTHELIAL_CELLS_MCPCOUNTER | 0.1722 | 0.0008 | 0.0222 | 1.0017 (1.0005, 1.0028) | 0.003579 | 1.0015 (1.0006, 1.0025) | 0.002216 |
| FIBROBLASTS_MCPCOUNTER | 0.1579 | 0.0021 | 0.0305 | 1.0000 (1.0000, 1.0000) | 0.045118 | 1.0000 (1.0000, 1.0000) | 0.133366 |
| ADC_XCELL | 0.0073 | 0.8875 | 0.9717 |  |  |  |  |
| ADIPOCYTES_XCELL | 0.0957 | 0.0638 | 0.1052 |  |  |  |  |
| ASTROCYTES_XCELL | 0.0523 | 0.3119 | 0.4197 |  |  |  |  |
| B.CELLS_XCELL | 0.058 | 0.2615 | 0.271 |  |  |  |  |
| BASOPHILS_XCELL | -0.1658 | 0.0012 | 0.0063 | 0.6445 (0.3903, 1.0640) | 0.085897 | 0.6985 (0.4588, 1.0634) | 0.094288 |
| CD4._MEMORY_T.CELLS_XCELL | 0.0968 | 0.0608 | 0.0359 |  |  |  |  |
| CD4._NAIVE_T.CELLS_XCELL | 0.0515 | 0.319 | 0.2028 |  |  |  |  |
| CD4._T.CELLS_XCELL | 0.1063 | 0.0393 | 0.063 | 1.0440 (0.0000, inf.) | 0.995964 | 76.9306 (0.0000, inf.) | 0.551673 |
| CD4._TCM_XCELL | 0.1248 | 0.0155 | 0.0694 | 7076.2170 (1.0759, inf.) | 0.048119 | 4654.0770 (2.3327, 9285415.8408) | 0.029369 |
| CD4._TEM_XCELL | 0.146 | 0.0046 | 0.0086 | 10.8389 (0.0180, 6516.5419) | 0.465418 | 5.4240 (0.0220, 1338.7900) | 0.54744 |
| CD8._NAIVE_T.CELLS_XCELL | -0.117 | 0.0233 | 0.1368 |  |  |  |  |
| CD8._T.CELLS_XCELL | 0.0282 | 0.5852 | 0.3972 |  |  |  |  |
| CD8._TCM_XCELL | 0.0749 | 0.1469 | 0.0769 |  |  |  |  |
| CD8._TEM_XCELL | 0.1308 | 0.0111 | 0.0168 | 1322.1272 (0.0000, inf.) | 0.565985 | 0.9503 (0.0000, inf.) | 0.996752 |
| CDC_XCELL | 0.1334 | 0.0096 | 0.0166 | 11.2232 (0.0659, 1910.7596) | 0.356256 | 3.2583 (0.0313, 339.3335) | 0.618246 |
| CHONDROCYTES_XCELL | 0.168 | 0.0011 | 0.0011 | 722.1975 (7.4484, 70024.1950) | 0.004797 | 250.2144 (4.1084, 15238.9330) | 0.008439 |
| CLASS.SWITCHED_MEMORY_B.CELLS_XCELL | 0.1021 | 0.048 | 0.0584 |  |  |  |  |
| CLP_XCELL | -0.1597 | 0.0019 | 0.0388 | 0.0001 (0.0000, 0.2274) | 0.020499 | 0.0002 (0.0000, 0.1920) | 0.014551 |
| CMP_XCELL | -0.1087 | 0.0352 | 0.031 | 0.0000 (0.0000, 3.8365) | 0.058717 | 0.0000 (0.0000, 747098.3601) | 0.320358 |
| DC_XCELL | 0.1294 | 0.012 | 0.0392 | 595540.0466 (0.0016, inf.) | 0.187104 | 2498.2581 (0.0000, inf.) | 0.40684 |
| ENDOTHELIAL_CELLS_XCELL | 0.2218 | 0 | 0.0003 | 484.9218 (3.9630, 59335.9416) | 0.011687 | 271.2917 (3.8479, 19127.3506) | 0.009863 |
| EOSINOPHILS_XCELL | 0.0125 | 0.8098 | 0.8321 |  |  |  |  |
| EPITHELIAL_CELLS_XCELL | 0.1145 | 0.0264 | 0.0733 |  |  | 13.0494 (0.0333, 5115.4486) | 0.39914 |
| ERYTHROCYTES_XCELL | -0.1687 | 0.001 | 0.0076 | 0.0001 (0.0000, 0.8657) | 0.046689 | 0.0003 (0.0000, 0.9481) | 0.048501 |
| FIBROBLASTS_XCELL | 0.0671 | 0.1945 | 0.0395 |  |  |  |  |
| GMP_XCELL | -0.0657 | 0.2039 | 0.0925 |  |  |  |  |
| HEPATOCYTES_XCELL | -0.1086 | 0.0353 | 0.044 | 0.0184 (0.0000, inf.) | 0.722773 | 0.0707 (0.0000, 9528772.4338) | 0.781478 |
| HSC_XCELL | 0.0167 | 0.7473 | 0.8915 |  |  |  |  |
| IDC_XCELL | 0.1401 | 0.0065 | 0.0171 | 7.2745 (0.3313, 159.7132) | 0.207993 | 2.9230 (0.1776, 48.1193) | 0.452926 |
| KERATINOCYTES_XCELL | 0.0831 | 0.1077 | 0.1765 |  |  |  |  |
| LY_ENDOTHELIAL_CELLS_XCELL | 0.1521 | 0.0031 | 0.0104 | inf. (0.6114, inf.) | 0.057034 | 2337639.4581 (0.7327, inf.) | 0.054948 |
| MACROPHAGES_XCELL | 0.1995 | 0.0001 | 0.0003 | 210.1227 (0.3788, 116543.8291) | 0.097136 | 16.5498 (0.0493, 5560.3990) | 0.344362 |
| MACROPHAGES_M1_XCELL | 0.1792 | 0.0005 | 0.002 | 2043.6624 (0.0944, inf.) | 0.134516 | 107.4428 (0.0136, 847122.5689) | 0.30695 |
| MACROPHAGES_M2_XCELL | 0.1713 | 0.0009 | 0.0019 | inf. (24.0023, inf.) | 0.014642 | 11965.4257 (0.0956, inf.) | 0.1169 |
| MAST_CELLS_XCELL | 0.1052 | 0.0415 | 0.1126 |  |  |  |  |
| MEGAKARYOCYTES_XCELL | 0.2074 | 0.0001 | 0.0003 | inf. (4938252.6813, inf.) | 0.028906 | inf. (30.3075, inf.) | 0.04394 |
| MELANOCYTES_XCELL | -0.1297 | 0.0118 | 0.0106 | 0.0000 (0.0000, 312.8113) | 0.117963 | 0.0000 (0.0000, 127.4665) | 0.118292 |
| MEMORY_B.CELLS_XCELL | 0.0034 | 0.9481 | 0.4124 |  |  |  |  |
| MEP_XCELL | 0.0685 | 0.185 | 0.1282 |  |  |  |  |
| MESANGIAL_CELLS_XCELL | 0.1468 | 0.0043 | 0.0626 |  |  |  |  |
| MONOCYTES_XCELL | 0.0914 | 0.0766 | 0.2222 |  |  |  |  |
| MPP_XCELL | -0.06 | 0.2457 | 0.3447 |  |  |  |  |
| MSC_XCELL | -0.1245 | 0.0157 | 0.0342 | 0.5220 (0.1839, 1.4819) | 0.222009 | 0.5969 (0.2481, 1.4358) | 0.249212 |
| MV_ENDOTHELIAL_CELLS_XCELL | 0.1992 | 0.0001 | 0.0033 | 237062.2053 (54.4292, inf.) | 0.003792 | 42543.5804 (23.1110, inf.) | 0.005458 |
| MYOCYTES_XCELL | -0.1657 | 0.0013 | 0.0018 | 0.0352 (0.0013, 0.9342) | 0.045423 | 0.0773 (0.0051, 1.1632) | 0.064216 |
| NAIVE_B.CELLS_XCELL | -0.0465 | 0.3686 | 0.2314 |  |  |  |  |
| NEURONS_XCELL | -0.1087 | 0.0351 | 0.0445 | 0.0000 (0.0000, 2.6482) | 0.073579 | 0.0005 (0.0000, 5.6047) | 0.110456 |
| NEUTROPHILS_XCELL | -0.0463 | 0.3709 | 0.8802 |  |  |  |  |
| NK_CELLS_XCELL | -0.0722 | 0.1621 | 0.2223 |  |  |  |  |
| NKT_XCELL | 0.0416 | 0.4217 | 0.85 |  |  |  |  |
| OSTEOBLAST_XCELL | 0 | 0.9997 | 0.8972 |  |  |  |  |
| PDC_XCELL | -0.0623 | 0.2284 | 0.7266 |  |  |  |  |
| PERICYTES_XCELL | 0.1743 | 0.0007 | 0.0043 | 69.5543 (4.6087, 1049.7160) | 0.002189 | 26.8689 (2.3780, 303.5947) | 0.007809 |
| PLASMA_CELLS_XCELL | 0.0263 | 0.6106 | 0.2162 |  |  |  |  |
| PLATELETS_XCELL | -0.018 | 0.7282 | 0.3631 |  |  |  |  |
| PREADIPOCYTES_XCELL | 0.0035 | 0.9455 | 0.9975 |  |  |  |  |
| PRO_B.CELLS_XCELL | -0.2279 | 0 | <0.0001 | 0.4487 (0.1872, 1.0751) | 0.072247 | 0.5390 (0.2602, 1.1167) | 0.096305 |
| SEBOCYTES_XCELL | 0.0519 | 0.3157 | 0.1676 |  |  |  |  |
| SKELETAL_MUSCLE_XCELL | -0.1389 | 0.007 | 0.0091 | 0.0234 (0.0005, 1.1161) | 0.056872 | 0.0538 (0.0022, 1.3276) | 0.073973 |
| SMOOTH_MUSCLE_XCELL | 0.2093 | 0 | 0.0002 | 2.7558 (1.0110, 7.5121) | 0.047555 | 2.2054 (0.9572, 5.0810) | 0.063262 |
| TGD_CELLS_XCELL | 0.0072 | 0.889 | 0.9072 |  |  |  |  |
| TH1_CELLS_XCELL | 0.088 | 0.0885 | 0.0882 |  |  |  |  |
| TH2_CELLS_XCELL | -0.2145 | 0 | 0.0014 | 0.0636 (0.0081, 0.5003) | 0.008843 | 0.0885 (0.0159, 0.4917) | 0.005588 |
| TREGS_XCELL | 0.1493 | 0.0037 | 0.0112 | 633.4908 (0.0000, inf.) | 0.517885 | 22991.4260 (0.0012, inf.) | 0.241323 |

HR, hazard ratio; CI, confidence interval; inf., infinity
